# Mendelian Randomization: A Review of Methods for the Prevention, Assessment, and Discussion of Pleiotropy in Studies Using the Fat Mass and Obesity-Associated Gene as an Instrument for Adiposity

**DOI:** 10.3389/fgene.2022.803238

**Published:** 2022-02-04

**Authors:** Fiston Ikwa Ndol Mbutiwi, Tatiana Dessy, Marie-Pierre Sylvestre

**Affiliations:** ^1^ University of Montreal Hospital Research Centre (CRCHUM), Montreal, QC, Canada; ^2^ Faculty of Medicine, University of Kikwit, Kikwit, Democratic Republic of the Congo; ^3^ Department of Social and Preventive Medicine, University of Montreal Public Health School (ESPUM), Montreal, QC, Canada

**Keywords:** Mendelian randomization, instrumental variable, pleiotropy, FTO, adiposity, body mass index, genetic risk score

## Abstract

Pleiotropy assessment is critical for the validity of Mendelian randomization (MR) analyses, and its management remains a challenging task for researchers. This review examines how the authors of MR studies address bias due to pleiotropy in practice. We reviewed Pubmed, Medline, Embase and Web of Science for MR studies published before 21 May 2020 that used at least one single*-*nucleotide polymorphism (SNP) in the fat mass and obesity-associated (FTO) gene as instrumental variable (IV) for body mass index, irrespective of the outcome. We reviewed: 1) the approaches used to prevent pleiotropy, 2) the methods cited to detect or control the independence or the exclusion restriction assumption highlighting whether pleiotropy assessment was explicitly stated to justify the use of these methods, and 3) the discussion of findings related to pleiotropy. We included 128 studies, of which thirty-three reported one approach to prevent pleiotropy, such as the use of multiple (independent) SNPs combined in a genetic risk score as IVs. One hundred and twenty studies cited at least one method to detect or account for pleiotropy, including robust and other IV estimation methods (*n* = 70), methods for detection of heterogeneity between estimated causal effects across IVs (*n* = 72), methods to detect or account associations between IV and outcome outside thought the exposure (*n* = 85), and other methods (*n* = 5). Twenty-one studies suspected IV invalidity, of which 16 explicitly referred to pleiotropy, and six incriminating *FTO* SNPs. Most reviewed MR studies have cited methods to prevent or to detect or control bias due to pleiotropy. These methods are heterogeneous, their triangulation should increase the reliability of causal inference.

## Introduction

Mendelian randomization (MR) is an instrumental variables (IVs) approach that exploits genetic variants (mostly single-nucleotide polymorphisms (SNPs)) as IVs of a non-genetic exposure to infer a causal relationship between this exposure and an outcome in observational studies (Lawlor et al., 2008). The validity of MR is based on three key assumptions: 1) the IV is associated with the exposure, 2) the association between the IV and the outcome is unconfounded, and 3) the IV only affects the outcome via the exposure, known as the exclusion restriction criterion (Labrecque and Swanson, 2018; Brumpton et al., 2020). Horizontal pleiotropy, the phenomenon whereby a genetic variant affects the exposure and the outcome through independent pathways and without being mediated by another (Davey Smith and Hemani, 2014; Hemani et al., 2018a; Jordan et al., 2019), is a primary cause of violation of the exclusion restriction criterion (Dixon et al., 2020). It may lead to biased causal effect estimates, reduced statistical power, and/or increased type I error (Bowden et al., 2016; Verbanck et al., 2018). We thereafter refer to “horizontal pleiotropy” as “pleiotropy” for the sake of brevity.

The increasing use of MR (Sekula et al., 2016) has prompted both subject-specific (Pingault et al., 2016; Frayling and Stoneman, 2018; Goodarzi, 2018; Lor et al., 2019; Meng et al., 2019; Guo et al., 2021) and general reviews (Bochud and Rousson, 2010; Davies et al., 2013; Boef et al., 2015) of MR studies summarizing the state of practice of MR in the last decade. These suggest that the exclusion restriction criterion is not systematically assessed or discussed. For example, in their meta-epidemiological overview on the approaches used in MR, Boef et al. (2015) noted that only 111 of 178 studies (62.4%) reported on the plausibility of the exclusion restriction criterion. However, no review thus far has focused on how authors prevent or minimize bias related to pleiotropy in MR studies. Such examination is important because while evidence suggests that pleiotropy is ubiquitous in the human genome (Boyle et al., 2017; Chesmore et al., 2018), the absence of pleiotropy in a MR study cannot be empirically proven (Glymour et al., 2012). While several approaches to detect pleiotropy and/or provide robust MR estimates have been recently proposed and compared (Bowden et al., 2015; Hartwig et al., 2017; Thompson et al., 2017; Hemani et al., 2018a; Verbanck et al., 2018; Rees et al., 2019; Burgess et al., 2020b; Zhao Q. et al., 2020; Minelli et al., 2021), their use in practice, including in sensitivity analyses and triangulation, has not been documented across studies. In light of the recent MR guidelines (Davey Smith et al., 2019; Burgess et al., 2020a) that recommend assessing the robustness of MR results, we examine how potential bias due to pleiotropy is considered in the literature. More specifically we summarize 1) the approaches used to avoid selecting pleiotropic genetic variants, 2) the methods used to detect and account for pleiotropy in the estimation of the causal effect, and 3) how researchers discuss the exclusion restriction criterion considering their assessment of pleiotropy, including the impact on the results when pleiotropy is suspected.

Reviewing the entire body of published MR studies would not be practical. Instead, we limited our investigation to MR studies that use SNPs in the fat mass and obesity*-*associated (*FTO*) gene as IV to investigate the causal effect of adiposity on diverse outcomes. We expected studies that used *FTO* as an IV to provide a rich discussion of pleiotropy for several reasons. First, several SNPs in *FTO* have large and robust associations with body mass index (BMI) (Gill et al., 2019) and thus are considered strong IVs for adiposity and commonly used in MR studies. Second, unlike IVs such as variants on the C-reactive protein gene which encodes C-reactive protein (Burgess et al., 2017; Reactive Protein Coronary Heart Disease Genetics Collaboration et al., 2011), the exact biological pathways through which *FTO* affect adiposity are not fully understood, which complexifies the assessment of pleiotropy. Third, some *FTO* SNPs are suspected of pleiotropy with reported effects on a wide array of health issues ranging from cardiometabolic outcomes to cancer or mental health (Pausova et al., 2009; Delahanty et al., 2011; Hertel et al., 2011; Kivimäki et al., 2011; Li et al., 2012; Iles et al., 2013; Liu et al., 2013; Cronin et al., 2014; Aijala et al., 2015).

## Methods

### Search Strategy and Inclusion/Exclusion Criteria

We searched Pubmed, Medline, Embase and Web of Science to identify articles published before 21 May 2020 that met the following three criteria: 1) the primary analysis was MR, 2) the primary exposure was adiposity assessed by BMI, and 3) the IV(s) included at least one SNP in the *FTO* gene. We excluded studies in which MR was a secondary analysis to ensure that the study provided a detailed assessment of MR assumptions. We placed no restrictions on the outcome of interest. The search strategy and the specific exclusion criteria are provided in Supplementary Methods S1, S2, respectively.

### Data Extraction and Analysis

For each study we recorded the use of a one-sample or two-sample MR design. W**e** considered as one-sample both 1) studies that performed MR using individual level data on the SNPs, exposure and outcome and 2) studies that used summary statistics on SNP-exposure and SNP-outcome associations from the same sample (Hemani et al., 2018a). Two-sample data were defined as the use of summary statistics on SNP-exposure and SNP-outcome associations both from two distinct samples (Hemani et al., 2018a). We documented three types of IVs including 1) single IV, 2) genetic risk scores (GRS) that aggregate several SNPs into a single variable that corresponds to a weighted or unweighted sum of risk alleles (Burgess and Thompson, 2013; Burgess et al., 2016) and 3) multiple IVs. We defined multiple IVs as the use of ≥2 SNPs as separate IVs in a single model or to the combination of estimated effects from ≥2 single SNPs into one summary causal effect using meta-analytic techniques (Burgess et al., 2016). We recorded the specific *FTO* SNP(s) used in each of the types of IVs described above.

We organized the data analysis around three themes that described how pleiotropy was handled in the analytical process including 1) the selection and combinations of IVs; 2) the methods used to detect and account for pleiotropy in the estimation of causal effects, and 3) the discussion of findings considering the assessment of pleiotropy. We documented the methods as they were explicitly stated in each article irrespective of their applicability or relevance. A critical appraisal of the use of some of the methods is provided in the Discussion.

#### Approaches to Prevent Pleiotropy (Selection and Combination of IVs)

According to MR guidelines (Burgess et al., 2020a), SNPs can either be selected from gene regions that specifically encodes the exposure (biological approach) or on the basis of their statistical association with the exposure of interest (statistical approach). SNPs known or suspected of pleiotropy may be excluded from the initial selection before performing the main analysis (Burgess et al., 2020a). We reported the use of the biological and or statistical approach to SNP selection. We also documented the use of multiple independent SNPs as IVs as a method to attenuate the effect of pleiotropy under the assumption that the pleiotropic effects of SNPs would be balanced and thus cancel each other out (Davey Smith, 2011). Finally, we recorded any other strategy explicitly presented as pertaining to the selection of IVs to minimize the presence of pleiotropy.

#### Approaches to Detect and/or Account for Pleiotropy in the Estimation of the Causal Effect

MR guidelines require authors to report on the methods used to evaluate MR assumptions, which includes investigating bias due to pleiotropy (Davey Smith et al., 2019; Burgess et al., 2020a). We recorded the methods used to evaluate the independence and the exclusion restriction assumptions with the exception of those used for population stratification, highlighting whether pleiotropy assessment was explicitly stated to justify the use of these methods. We organized methods into four categories including 1) robust (e.g., MR-Egger) and other IV estimation methods (e.g., multivariable MR), 2) methods to detect heterogeneity of estimated causal effects across IVs (e.g., statistical tests of heterogeneity between the estimated SNP-specific causal effects), 3) methods to detect or account for associations between the IVs and the outcome that arise through pathways outside of the exposure (e.g., mediation analysis), and 4) other methods. Robust methods provide causal effect estimates under a weaker set of assumptions than conventional methods (Burgess and Thompson, 2017; Burgess et al., 2020a). A summary description of the methods, including their main assumptions and limitations is presented in Supplementary Table S6.

#### Discussion of Findings Considering the Assessment of Pleiotropy

We verified whether the authors discussed the independence and the exclusion restriction assumptions, distinguishing studies that explicitly referred to the term “pleiotropy” from those that did not. When studies suspected IV invalidity, we further verified whether the authors report the impact of IV invalidity on MR results, and if any *FTO* SNPs were incriminated.

The data were tabulated using Microsoft Excel^®^ 2016 and described using Stata/IC version 14.2 software (StataCorp, College Station, Texas, United States).

## Results

### Study Selection and Characteristics of Studies

**FIGURE 1:**
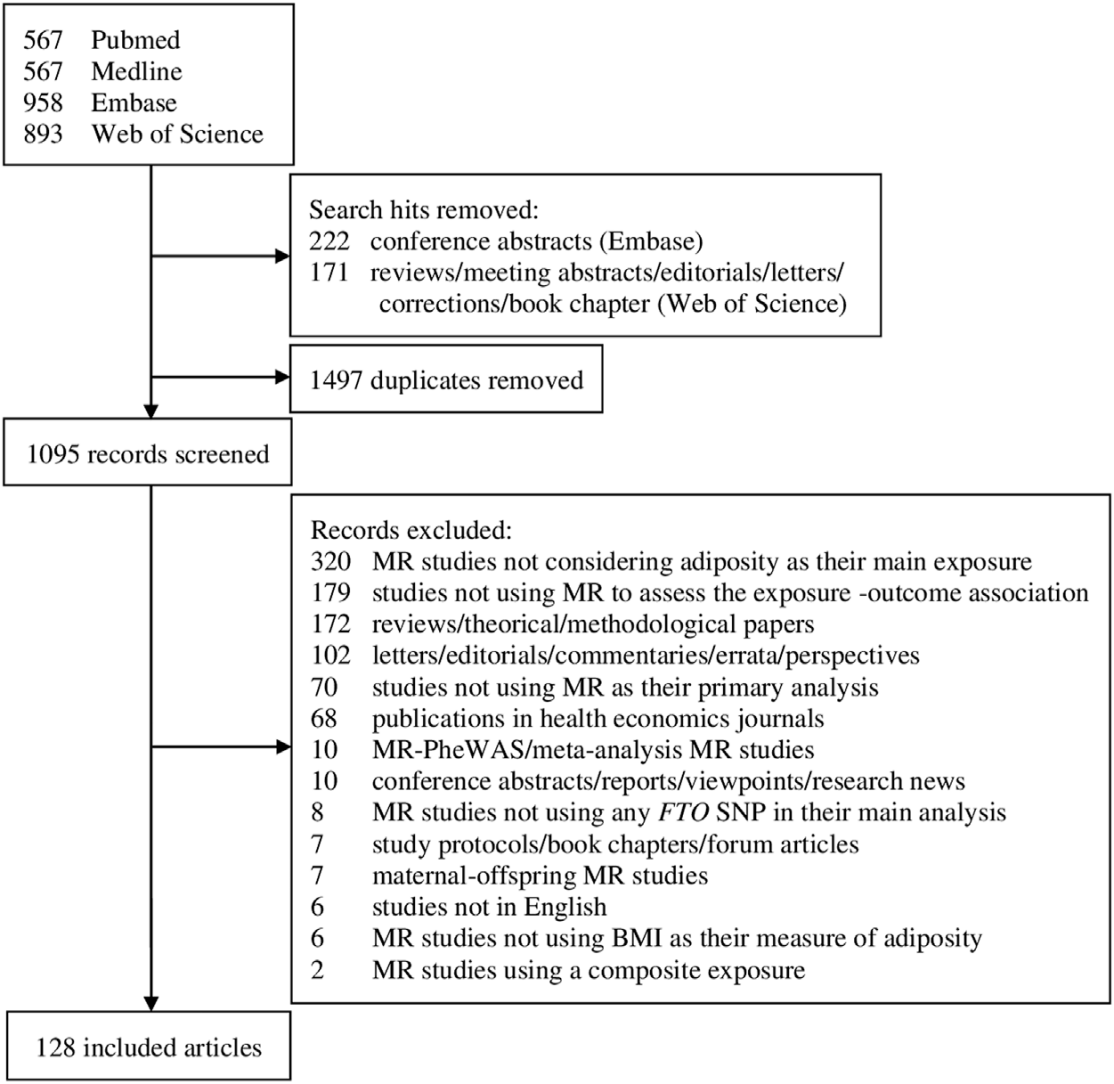
Flowchart of selection of studies includes in the review. MR, Mendelian randomization; PheWAS, phenome-wide association study; *FTO*, fat mass and obesity-associated; SNP, single*-*nucleotide polymorphism; BMI, body mass index.

**FIGURE 2:**
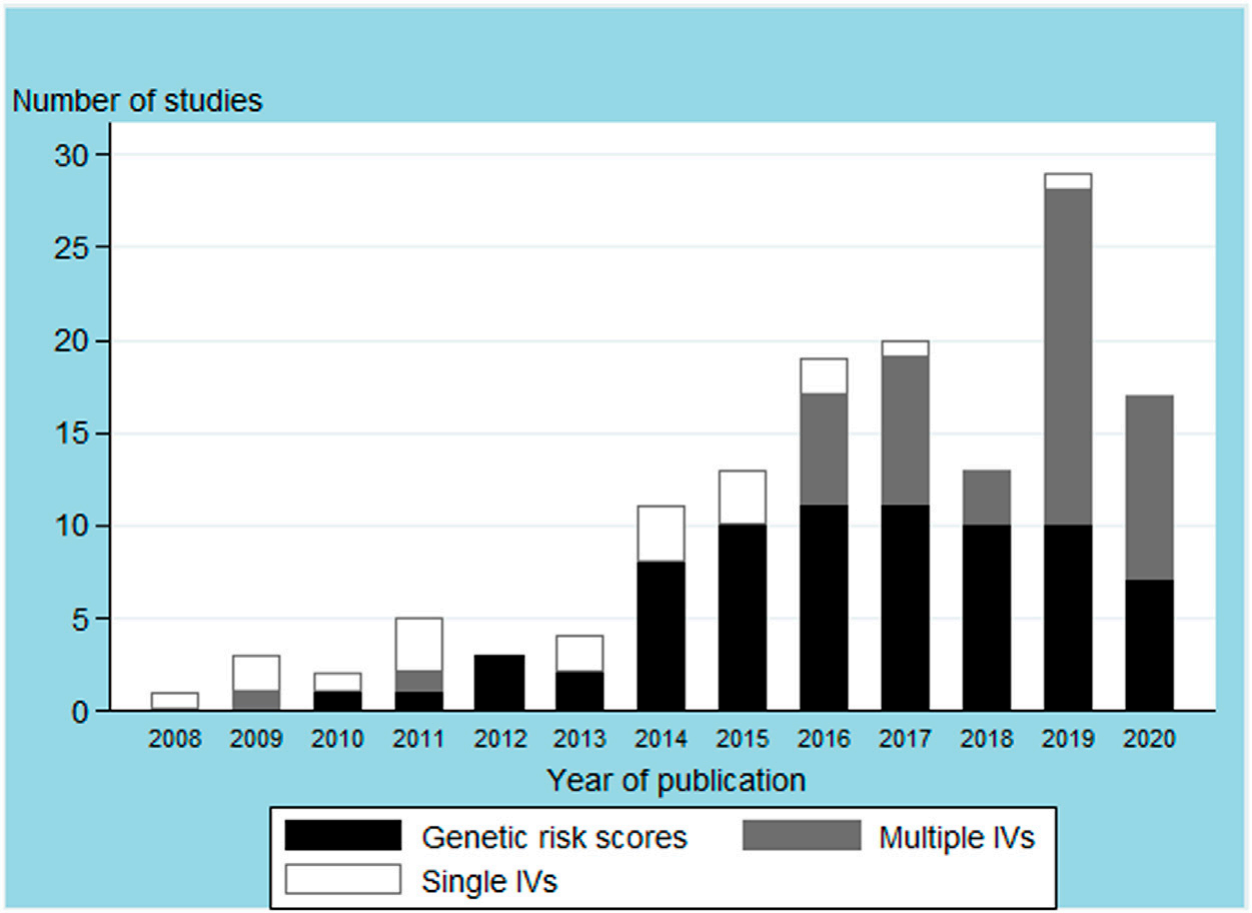
Body mass index instrumental variables (IVs) used in the main analysis by article publication year.

Our search identified 2,985 publications, of which 128 articles were included upon completion of the screening process (Figure 1). The 128 articles are listed in Supplementary Table S1. Included articles were published between June 2008 and May 2020, and mostly comprised one-sample MR analyses (*n* = 98, 76.6%; Table 1 and Supplementary Table S3). A total of 31 *FTO* SNPs were selected as IVs with rs1558902 being the most frequently used (*n* = 64, 50%; Supplementary Table S2). While 74 studies (57.8%) used a GRS to represent the IVs (Table 1 and Supplementary Table S3 **)**, Figure 2 suggests a recent decline in the use of GRS in favor of multiple IVs.

**TABLE 1:** Characteristics of included studies.

Characteristics of studies	*n*	%
Types of data used	*n* = 128	
One-sample only	82	64.1
Two-sample only	30	23.4
Both one and two samples	16	12.5
Types of instruments used in the main analysis	*n* = 128	
GRS only	63	49.2
Multiple IVs only	40	31.2
Single IVs only	13	10.2
Both GRS and multiple IVs	6	4.7
Both GRS and single IVs	5	3.9
Both single and multiple IVs	1	0.8
Types of GRS used among studies using GRS as IV in the main analysis	*n* = 74	
Weighted GRS only	48	64.9
Unweighted GRS only	19	25.7
Both weighted and unweighted GRS	7	9.4

Abbreviations: GRS, genetic risk score; IVs, instrumental variables; SNPs, single*-*nucleotide polymorphisms; *FTO*, fat mass and obesity-associated.

### Approaches to Prevent Pleiotropy (Selection and Combination of IVs)

While all of the 128 studies selected IVs on the basis of statistical association between SNPs and BMI in the literature, 33 studies (25.8%) proceeded further in their attempt to prevent the effect of pleiotropy in the selection of the IVs (Table 2 and Supplementary Table S4). Of the 33 studies, 12 excluded previously selected SNPs known or suspected of pleiotropy. Ten articles respectively cited the use of a GRS and of multiple (independent) IVs (even if six of these ten in fact used a GRS as IVs) to prevent the effect of pleiotropy, a single study justified the use of a single SNP as IV as a method to prevent pleiotropy, although these strategies do not guarantee that bias due to pleiotropy is prevented or reduced (Burgess and Thompson, 2013).

**TABLE 2:** Approaches cited for preventing pleiotropy in the selection of IVs.

Approaches	*n*/33	%
Exclude selected SNPs known or suspected to be pleiotropic	12	36.4
Use of multiple (independent) SNPs combined in a GRS as IVs	10	30.3
Use of multiple (independent) SNPs as IVs	10^a^	30.3
Use a single SNP as IV	1	3.0

Abbreviations: GRS, genetic risk score; SNP, single*-*nucleotide polymorphisms; IV, instrumental variable.

a These 10 articles discussed the use of multiple independent SNPs without explicitly mentioning GRS even if six of the 10 in fact used a GRS as IV in the main analysis.

### Approaches to Detect and Account for Pleiotropy in the Estimation of the Causal Effect

Overall, 120 of 128 included studies assessed the plausibility of the independence and/or exclusion restriction assumptions (Table 3 and Supplementary Table S5). Of the 120 studies, 78 reported using more than one category of methods within our classification (robust and other IV estimation methods (*n* = 70), heterogeneity (*n* = 72), alternative pathways (*n* = 85) and others (*n* = 5, including the use of positive or negative control outcomes, colocalization, and verifying the concordance of MR results with those from other studies). A total of 95 studies explicitly cited pleiotropy to justify the use of such methods. MR-Egger was the most frequently reported method to assess pleiotropy (*n* = 68). Of the 68 studies, 66 used the intercept *p*-value as a test of the validity of IVs and 29 studies (Gao et al., 2016; Hartwig et al., 2016; Kemp et al., 2016; Mokry et al., 2016; Tyrrell et al., 2016; Censin et al., 2017; Dale et al., 2017; Lindstrom et al., 2017; Lyall et al., 2017; Noyce et al., 2017; Xu et al., 2017; Larsson et al., 2018; van den Broek et al., 2018; Wang T. et al., 2018; Bae and Lee, 2019; Brower et al., 2019; Censin et al., 2019; Gharahkhani et al., 2019; Howe et al., 2019; Taylor et al., 2019; Tyrrell et al., 2019; Xu et al., 2019; Dusingize et al., 2020; Kurz and Laxy, 2020; Larsson et al., 2020; Liu et al., 2020; Winter-Jensen et al., 2020; Zhang et al., 2020; Zhao Y. et al., 2020) further acknowledged that MR-Egger estimates or tests may be underpowered. Two studies (Tyrrell et al., 2016; Fan et al., 2018) compared the MR-Egger slope and the conventional MR causal effect estimate while the last study (Chen et al., 2019) did not specify how the MR-Egger results were used. Forty-six studies assessed pleiotropy by evaluating the heterogeneity of estimated causal effects across IVs, whether by graphical assessment (*n* = 20) or statistical testing (*n* = 7), or by comparing estimated causal effects from GRS or multiple IVs before vs. after exclusion of suspected pleiotropic SNP(s) (*n* = 20). A total of 30 studies attempted to detect pleiotropy by estimating pathways through which the IVs were associated with the outcomes outside of that implicating the exposure. Such studies mostly reported the estimation of associations between the IVs and measured risk factors of the outcome (*n* = 19), but also adjusted the IV-outcome or IV-confounders associations for exposure (*n* = 7) or documented the associations between the IV and risk factors for the outcome in the literature (*n* = 5).

**TABLE 3:** Methods cited for detection or accounting the violation of the independence and/or restriction exclusion assumptions.

Methods^a^	*n*/128	Studies specifically referred to the method for “pleiotropy” assessment/control/robustness (*n* = 95)	Studies not specifically referred to the method for “pleiotropy” assessment/control/robustness (*n* = 96)
Robust and other IV estimation methods^b^	70	69	23
Robust methods	69	68	20
MR-Egger	69^c^	68	1
Median-based methods	44	26	18
Mode-based methods	9	5	4
MR-PRESSO	6	5	1
MR-RAPS	1	0	1
Other IV estimation methods	13	6	8
Multivariable MR	6	3	3
IVW methods	4	3	1
Likelihood-based methods	4	0	4
Methods to detect heterogeneity of estimated causal effects across IVs	72	46	46
Graphical assessment: scatter plots, forest plots, funnel plots, leave-one-out plots, and histogram	34	20	19
Statistical criteria and tests: I^2^, r^2^, H statistic, Cochran’s Q, Rucker’s Q, over-identification tests^d^	30	7	23
Comparisons of estimated MR causal effects across IVs (GRS or multiple IVs)	39	25	14
Before and after exclusion of SNPs suspected of pleiotropy	33	20	13
GRS or multiple IVs vs. single SNP(s)	5	5	0
Two subsets of SNPs grouping SNPs with the same biological pathway on the exposure	1	0	1
Detection of outlier/influential SNP(s): Cook’s distance, Studentized residuals, HEIDI-outlier, leave-one-out analyses	15	4	13
Methods to detect associations between IVs and the outcome outside of the pathway through the exposure	85	30	61
Estimating the associations between the IVs and measured risk factors for the outcome	75	19	56
Documenting the associations between the IV and risk factors for the outcome in the literature	7	5	2
Adjusting IV-outcome or IV-confounders associations for exposure	18	7	11
Adjusting IV-outcome association or MR analyses for covariates potentially involved in pleiotropic pathways^e^	5	2	3
Comparison of the exposure-outcome conventional vs. IV estimated effects	4	1	3
Comparison of the IV-outcome vs. IV-exposure associations	2	2	0
Estimating the association between the IV and the outcome	2	1	1
Mediation analysis estimating the direct effect of the IV on the outcome	3	0	3
Other methods	5	0	5
The use of positive or negative control outcomes	3	0	3
Colocalization	1	0	1
Verifying the concordance of MR results with those from other studies (MR, clinical trial)	1	0	1
No method reported for assessment of independence or exclusion restriction assumptions	8		

Abbreviations: IV, instrumental variable; MR, Mendelian randomization; PRESSO, pleiotropy residual sum and outlier; SNP, single*-*nucleotide polymorphism; RAPS, Robust Adjusted Prole Score; IVW, inverse-variance weighted; *FTO*, fat mass and obesity-associated; GRS, genetic risk score; HEIDI, Heterogeneity in Dependent Instruments.

a Many studies cited more than one method and thus the number of methods reported in the Table exceed the total number of studies (*n* = 128).

b See (Burgess et al., 2020a) and (Burgess et al., 2015) for a summary of the listed methods.

c Of the 69 studies that reported using MR-Egger, 66 used the intercept test *p*-value to infer whether or not pleiotropy was present, two studies (Tyrrell et al., 2016; Fan et al., 2018) compared the MR-Egger slope and the conventional MR causal effect estimate, while the last study (Chen et al., 2019) did not specify how the MR-Egger results were used.

d One study (Censin et al., 2017) did not specify the heterogeneity test used.

e Three articles (Guo et al., 2016; Censin et al., 2019; Sun et al., 2020) mentioned adjustment of MR analyses for covariates potentially involved in pleiotropic pathways without specifying as multivariable MR.

### Discussion of Findings Considering the Assessment of Pleiotropy

Of 128 included articles, 108 discussed the plausibility of the independence and/or exclusion restriction assumptions of which 89 studies made an explicit reference to pleiotropy (Table 4 and Supplementary Table S7). Invalid IVs were suspected in 21 studies (Supplementary Table S8), 16 of which cited pleiotropy as a potential source of invalidity. Eight of the 21 studies concluded that the MR results were possibly invalid, while nine studies reported that the results were robust. The remaining four studies did not discuss the impact of IV invalidity on the results. Six studies suggested that at least one *FTO* SNP (rs1558902, rs1421085, and rs17817449) was suspected of pleiotropy on the basis of sensitivity analyses (Table 4, Supplementary Tables S7, S8).

**TABLE 4:** Discussion of the independence and/or exclusion restriction assumptions.

Discussion of the independence and/or exclusion restriction assumptions	*n*	%
Plausibility of the independence and/or exclusion restriction assumptions	*n* = 128	
Discussed with specific reference to pleiotropy	89	69.5
Discussed without specific reference to pleiotropy	19	14.9
IV invalidity^a^	*n* = 128	
Suspected with specific reference to pleiotropy	16	12.5
Suspected without specific reference to pleiotropy	5	3.9
Impact of IV invalidity on the validity of MR results	*n* = 21	
May have affected validity of results	8	38.1
No or low impact on validity of results	9	42.9
Impact not (clearly) reported	4	19.0
Suspicion of invalidity/pleiotropy of *FTO* SNP(s)	*n* = 128	
Yes	6	4.7
*FTO* SNP(s) suspected to be invalid/pleiotropic	*n* = 6	
rs1558902^b^	3	50.0
rs1421085^c^	2	33.3
rs17817449^d^	1	16.7

Abbreviations: IV, instrumental variable; MR, Mendelian randomization; SNP, single*-*nucleotide polymorphism; *FTO*, fat mass and obesity-associated.

a Refers to the suspicion of invalidity of one or more body mass index IV(s) for any outcome of interest.

b The outcomes of interest involved in the suspected invalidity of rs1558902 were multiple sclerosis susceptibility (Gianfrancesco et al., 2017), phobic anxiety symptoms (Walter et al., 2015a), and depression (Walter et al., 2015b).

c The outcomes of interest involved in the suspected pleiotropy of rs1421085 were common mental disorders (Kivimäki et al., 2011), and subjective well-being (van den Broek et al., 2018).

d The outcome of interest involved in the suspected pleiotropy of rs17817449 was lipid profiles (Wang N. et al., 2018).

## Discussion

Pleiotropy is considered widespread in humans (Boyle et al., 2017) and thus presents a major challenge to the validity of MR studies, especially considering the limited knowledge of the biological function of many of the SNPs used as IVs (Danchin and Fang, 2016; Swerdlow et al., 2016). We reviewed studies that used SNP(s) in the *FTO* gene as IV(s) in MR to examine the strategies employed by authors to prevent, detect or control, and discuss biases due to the use of pleiotropic IVs. Our review extends the overview of statistical approaches used in MR published by Boef et al. (2015) by focusing on pleiotropy and by including the recent developments such as two-sample MR and the use of MR-Egger. We observed that the vast majority of studies addressed pleiotropy by using several methods that operate under different assumptions (Lawlor et al., 2016; Hemani et al., 2018b). While most authors invoked pleiotropy at the analytical stage to justify the use of detection tools and robust methods, explicit attention was also given to the prevention of pleiotropy in the selection of IVs in a fourth of the articles reviewed and pleiotropy was mentioned in the discussion in 70% of articles.

Our review highlighted three observations that merit attention for future MR studies. First, we documented an increasing use of multiple IVs over time, in addition to the exclusive use of statistical criteria to select IVs from the literature. This is largely explained by our focus on BMI as the exposure, since BMI is a polygenetic trait without a specific proximal coding gene from which to select SNPs as it is common with protein-like exposures (Swerdlow et al., 2016; Burgess and Thompson, 2017). The increasing use of multiple SNPs is also motivated by attempts to increase the strength of the instruments, the availability of large-scale genome-wide association studies to select instruments from, and by the development of MR methods that require multiple IVs to provide robust MR estimates under a less stringent set of assumptions (Burgess and Thompson, 2017; Burgess et al., 2020a). However, selecting SNPs exclusively by statistical approach increases the likelihood of including pleiotropic SNPs which may lead to biased MR results (Hartwig et al., 2017; Bowden and Holmes, 2019). Advantages of using multiple IVs independently in a MR study include the use of robust methods, such as MR-Egger or median or mode-based methods (Slob and Burgess, 2020). On the other hand, the use of GRS, which was the most frequent method to combine SNPs in the studies that we reviewed, is convenient because it leads to a single IV (Burgess et al., 2016). Further, weighted GRS with independently-derived weights lead to MR studies with similar statistical power than those using multiple independent IVs (Palmer et al., 2012). While several studies justified the use of a GRS as IV as a method to prevent bias due to pleiotropy, they have to rely on the restrictive assumption that the pleiotropic effects of SNPs cancel each other (Davey Smith, 2011), which is difficult to verify in practice. Using a GRS as IV further requires ensuring that each SNP in the GRS is itself a valid IV (Burgess and Thompson, 2013; Skaaby et al., 2018), which is limited by the low statistical power available for each SNP. Simulation studies have demonstrated that even including a small number of pleiotropic SNPs into a GRS can lead to biased MR estimates (Burgess and Thompson, 2013). We thus recommend that robust methods be used on the SNPs that form the GRS.

Second, our review suggests that the MR-Egger intercept test from is the most frequently reported method for pleiotropy assessment. The validity of MR-Egger estimates and the interpretation of the intercept as the average pleiotropic effect of IVs require the InSIDE (Instrument Strength Independent of Direct Effect) assumption to be satisfied (Burgess and Thompson, 2017). InSIDE is not required for the use of the *p*-value associated with the intercept test of the validity of IVs (Burgess and Thompson, 2017). InSIDE states that the effects of the IVs on the exposure must be uncorrelated with the direct effects of the IVs on the outcome (Bowden et al., 2015; Burgess et al., 2017), which is likely to be violated in a one-sample setting (Slob et al., 2017; Minelli et al., 2021) because parameters are estimated in the same subjects. Violations of the InSIDE assumption results in increased type I error rates (Hartwig and Davies, 2016; Burgess et al., 2017) and biased estimates in the direction of the observational associations (Minelli et al., 2021). Because testing the plausibility of InSIDE assumption is still a challenge to date (Bowden, 2017), researchers should restrict the interpretation of MR-Egger estimates in two sample settings where the lack of correlation between SNPs-exposure and SNPs-outcome associations is more plausible. Thus, when using summarized data in a one-sample setting, the MR-Egger intercept test can be used to assess the invalidity of the IVs, but other robust methods such as the median (the second robust method widely reported in this review)- and the mode-based methods should be preferred when estimating the robust causal estimates because they do not depend on the InSIDE assumption (Bowden, 2017). Further, their causal estimates are consistent in one-sample context, unlike Egger’s estimates which are biased in the direction of the observational association, as shown in simulations (Minelli et al., 2021). Burgess and Thompson offer a careful discussion of the use of MR-Egger (Burgess and Thompson, 2017), while Burgess et al. (2018) show that the statistical power for the intercept test is low in most settings.

Third, using statistical approaches to detect pleiotropic IVs is challenging because apparent manifestations of pleiotropy may be confused with other phenomena, some of which not invalidating MR or requiring different approaches than pleiotropy. For example, the assessment of heterogeneity in the estimated causal effects across different IVs is based on the principle that if IVs are valid, the variation in their corresponding MR estimates should be due to chance (Greco et al., 2015). Large variations in MR IV-specific estimates are often considered as indicative of pleiotropy, but can be due to other causes such as the non-collapsibility of odds ratios in case of MR analysis with binary outcome (Vansteelandt et al., 2011; Hemani et al., 2018a), heterogeneity in the distribution of confounders of IV-exposure or IV-outcome associations in two-sample settings (Hemani et al., 2018a; Zhao Q. et al., 2020), or differential complier causal effects, i.e., association between the IVs and the exposure that vary importantly across individuals (Baiocchia et al., 2014; Sainani, 2018). Similarly, reasons other than pleiotropy may explain non-null associations between the IVs and the outcome that may be considered indicative of pleiotropic IVs. For example, population stratification which can be addressed by restricting the sample to homogeneous ancestry or by applying correction methods (e.g., adjustment of MR models for principal components) (Davey Smith and Hemani, 2014). Additional causes of violation of the exclusion restriction that can be confused with pleiotropy have been proposed, including an exposure that varies over time, the presence of gene-environment interactions implicating IVs, and linkage disequilibrium between at least one of the IVs and a SNP that also affects the outcome (VanderWeele et al., 2014).

Our review also allows a few observations pertaining to the use of SNPs in the *FTO* gene as IVs for BMI. Four of the six studies that suspected that *FTO* SNPs used as IVs might be pleiotropic involved mental health phenotypes [e.g., subjective well-being (van den Broek et al., 2018) or phobic anxiety symptoms (Walter et al., 2015a), or common mental disorders (Kivimäki et al., 2011), including depression (Walter et al., 2015b)]. This suggests that *FTO* may be associated with mental health through pathways that do not involve BMI, a hypothesis that is supported by animal studies (Hess et al., 2013; Sun et al., 2019). For example, *FTO* regulates the activity of the dopaminergic signaling pathways related to the regulation of learning, reward behavior, motor functions, and feeding in mice (Hess et al., 2013). Furthermore, other work on *FTO*-deficient mice suggested that *FTO* could influence anxiety- and depression-like behaviours via alterations in gut microbiota (Sun et al., 2019). Caution is required regarding the use of *FTO* as an IV for BMI implicating mental health phenotypes.

Two limitations of the current review should be noted. First, we do not present an exhaustive list of the methods to prevent, detect or control, and discuss biases due to the use of pleiotropic IVs. Rather we focus on the methods reported in the 128 studies that we review. While we captured most of the methods that are currently available, newer methods such as the Causal Analysis Using Summary Effect estimates (CAUSE) (Morrison et al., 2020) and MR analysis using mixture-model (MRMix) (Qi and Chatterjee, 2019) are not reported. Second, the methods reported in this review include common and validated methods, as well as methods or strategies that may be less efficient/optimal for detecting or accounting bias due to pleiotropy in MR studies. Users must exert caution in selecting the best method(s) for the data at hand.

Pleiotropy is a ubiquitous phenomenon that poses a threat to the validity of MR results and that is difficult to assess. MR-related methodological development is thriving, and users are encouraged to use more than one method to assess pleiotropy, heeding the assumptions required for each.

## References

[B1] Aijala M. Ronkainen J. Huusko T. Malo E. Savolainen E.-R. Savolainen M. J. (2015). The Fat Mass and Obesity-Associated (FTO) Gene Variant Rs9939609 Predicts Long-Term Incidence of Cardiovascular Disease and Related Death Independent of the Traditional Risk Factors. Ann. Med. 47, 655–663. 10.3109/07853890.2015.1091088 26555680

[B2] Bae S. C. Lee Y. H. (2019). Causal Association between Body Mass index and Risk of Rheumatoid Arthritis: A Mendelian Randomization Study. Eur. J. Clin. Invest. 49, e13076. 10.1111/eci.13076 30710354

[B3] Baiocchi M. Cheng J. Small D. S. (2014). Instrumental Variable Methods for Causal Inference. Statist. Med. 33, 2297–2340. 10.1002/sim.6128 PMC4201653 24599889

[B4] Bochud M. Rousson V. (2010). Usefulness of Mendelian Randomization in Observational Epidemiology. Ijerph 7, 711–728. 10.3390/ijerph7030711 20616999 PMC2872313

[B5] Boef A. G. C. Dekkers O. M. le Cessie S. (2015). Mendelian Randomization Studies: a Review of the Approaches Used and the Quality of Reporting. Int. J. Epidemiol. 44, 496–511. 10.1093/ije/dyv071 25953784

[B6] Bowden J. Davey Smith G. Burgess S. (2015). Mendelian Randomization with Invalid Instruments: Effect Estimation and Bias Detection through Egger Regression. Int. J. Epidemiol. 44, 512–525. 10.1093/ije/dyv080 26050253 PMC4469799

[B106] Bowden J. Del Greco M. F. Minelli C. Davey Smith G. Sheehan N. A. Thompson J. (2016). Assessing the Suitability of Summary data for Two-Sample Mendelian Randomization Analyses Using MR-Egger Regression: The Role of the I 2 Statistic. Int. J. Epidemiol. 45, 1961–1974. 10.1093/ije/dyw220 27616674 PMC5446088

[B7] Bowden J. Holmes M. V. (2019). Meta‐analysis and Mendelian Randomization: A Review. Res. Syn Meth 10, 486–496. 10.1002/jrsm.1346 PMC6973275 30861319

[B8] Bowden J. (2017). Misconceptions on the Use of MR-Egger Regression and the Evaluation of the InSIDE assumption. Int. J. Epidemiol. 46, 2097–2099. 10.1093/ije/dyx192 29025021 PMC5837449

[B9] Boyle E. A. Li Y. I. Pritchard J. K. (2017). An Expanded View of Complex Traits: from Polygenic to Omnigenic. Cell 169, 1177–1186. 10.1016/j.cell.2017.05.038 28622505 PMC5536862

[B10] Brower M. A. Hai Y. Jones M. R. Guo X. Chen Y.-D. I. Rotter J. I. (2019). Bidirectional Mendelian Randomization to Explore the Causal Relationships between Body Mass index and Polycystic Ovary Syndrome. Hum. Reprod. 34, 127–136. 10.1093/humrep/dey343 30496407 PMC6295958

[B11] Brumpton B. Sanderson E. Heilbron K. Hartwig F. P. Harrison S. Vie G. A. (2020). Avoiding Dynastic, Assortative Mating, and Population Stratification Biases in Mendelian Randomization through Within-Family Analyses. Nat. Commun. 11, 3519. 10.1038/s41467-020-17117-4 32665587 PMC7360778

[B12] Burgess S. Bowden J. Dudbridge F. Thompson S. G. (2018). Assessing the Effectiveness of Robust Instrumental Variable Methods Using Multiple Candidate Instruments with Application to Mendelian Randomization. arXiv, 1606.03729v03722.

[B13] Burgess S. Bowden J. Fall T. Ingelsson E. Thompson S. G. (2017). Sensitivity Analyses for Robust Causal Inference from Mendelian Randomization Analyses with Multiple Genetic Variants. Epidemiology 28, 30–42. 10.1097/EDE.0000000000000559 27749700 PMC5133381

[B14] Burgess S. Davey Smith G. Davies N. M. Dudbridge F. Gill D. Glymour M. M. (2020a). Guidelines for Performing Mendelian Randomization Investigations. Wellcome Open Res. 4, 186. 10.12688/wellcomeopenres.15555.2 32760811 PMC7384151

[B15] Burgess S. Dudbridge F. Thompson S. G. (2016). Combining Information on Multiple Instrumental Variables in Mendelian Randomization: Comparison of Allele Score and Summarized Data Methods. Statist. Med. 35, 1880–1906. 10.1002/sim.6835 PMC4832315 26661904

[B16] Burgess S. Foley C. N. Allara E. Staley J. R. Howson J. M. M. (2020b). A Robust and Efficient Method for Mendelian Randomization with Hundreds of Genetic Variants. Nat. Commun. 11, 376. 10.1038/s41467-019-14156-4 31953392 PMC6969055

[B17] Burgess S. Scott R. A. Scott R. A. Timpson N. J. Davey Smith G. Thompson S. G. Epic- InterAct Consortium (2015). Using Published Data in Mendelian Randomization: a Blueprint for Efficient Identification of Causal Risk Factors. Eur. J. Epidemiol. 30, 543–552. 10.1007/s10654-015-0011-z 25773750 PMC4516908

[B18] Burgess S. Thompson S. G. (2017). Interpreting Findings from Mendelian Randomization Using the MR-Egger Method. Eur. J. Epidemiol. 32, 377–389. 10.1007/s10654-017-0255-x 28527048 PMC5506233

[B19] Burgess S. Thompson S. G. (2013). Use of Allele Scores as Instrumental Variables for Mendelian Randomization. Int. J. Epidemiol. 42, 1134–1144. 10.1093/ije/dyt093 24062299 PMC3780999

[B20] Censin J. C. Nowak C. Cooper N. Bergsten P. Todd J. A. Fall T. (2017). Childhood Adiposity and Risk of Type 1 Diabetes: A Mendelian Randomization Study. Plos Med. 14, e1002362. 10.1371/journal.pmed.1002362 28763444 PMC5538636

[B21] Censin J. C. Peters S. A. E. Bovijn J. Ferreira T. Pulit S. L. Mägi R. (2019). Causal Relationships between Obesity and the Leading Causes of Death in Women and Men. Plos Genet. 15, e1008405. 10.1371/journal.pgen.1008405 31647808 PMC6812754

[B22] Chen Y.-C. Fan H.-Y. Yang C. Hsieh R.-H. Pan W.-H. Lee Y. L. (2019). Assessing Causality between Childhood Adiposity and Early Puberty: A Bidirectional Mendelian Randomization and Longitudinal Study. Metabolism 100, 153961. 10.1016/j.metabol.2019.153961 31422054

[B23] Chesmore K. Bartlett J. Williams S. M. (2018). The Ubiquity of Pleiotropy in Human Disease. Hum. Genet. 137, 39–44. 10.1007/s00439-017-1854-z 29164333

[B24] Cronin R. M. Field J. R. Bradford Y. Shaffer C. M. Carroll R. J. Mosley J. D. (2014). Phenome-wide Association Studies Demonstrating Pleiotropy of Genetic Variants within FTO with and without Adjustment for Body Mass index. Front. Genet. 5, 250. 10.3389/fgene.2014.00250 25177340 PMC4134007

[B25] Dale C. E. Fatemifar G. Palmer T. M. White J. Prieto-Merino D. Zabaneh D. (2017). Causal Associations of Adiposity and Body Fat Distribution with Coronary Heart Disease, Stroke Subtypes, and Type 2 Diabetes Mellitus. Circulation 135, 2373–2388. 10.1161/CIRCULATIONAHA.116.026560 28500271 PMC5515354

[B26] Danchin A. Fang G. (2016). Unknown Unknowns: Essential Genes in Quest for Function. Microb. Biotechnol. 9, 530–540. 10.1111/1751-7915.12384 27435445 PMC4993169

[B27] Davey Smith G. Davies N. M. Dimou N. Egger M. Gallo V. Golub R. (2019). STROBE-MR: Guidelines for Strengthening the Reporting of Mendelian Randomization Studies. PeerJ Preprints 7, e27857v27851. 10.7287/peerj.preprints.27857v1

[B28] Davey Smith G. Hemani G. (2014). Mendelian Randomization: Genetic Anchors for Causal Inference in Epidemiological Studies. Hum. Mol. Genet. 23, R89–R98. 10.1093/hmg/ddu328 25064373 PMC4170722

[B29] Davey Smith G. (2011). Random Allocation in Observational Data. Epidemiology 22, 460–463. 10.1097/EDE.0b013e31821d0426 21642771

[B30] Davies N. M. Smith G. D. Windmeijer F. Martin R. M. (2013). Issues in the Reporting and Conduct of Instrumental Variable Studies. Epidemiology 24, 363–369. 10.1097/EDE.0b013e31828abafb 23532055

[B31] Delahanty R. J. Beeghly-Fadiel A. Xiang Y.-B. Long J. Cai Q. Wen W. (2011). Association of Obesity-Related Genetic Variants with Endometrial Cancer Risk: a Report from the Shanghai Endometrial Cancer Genetics Study. Am. J. Epidemiol. 174, 1115–1126. 10.1093/aje/kwr233 21976109 PMC3246689

[B105] Dixon P. Hollingworth W. Harrison S. Davies N. M. Davey Smith G. (2020). Mendelian Randomization analysis of the causal effect of adiposity on hospital costs. J. Health Econ. 70, 102300. 10.1016/j.jhealeco.2020.102300 32014825 PMC7188219

[B32] Dusingize J. C. Olsen C. M. An J. Pandeya N. Law M. H. Thompson B. S. (2020). Body Mass index and Height and Risk of Cutaneous Melanoma: Mendelian Randomization Analyses. Int. J. Epidemiol. 49, 1236–1245. 10.1093/ije/dyaa009 32068838 PMC7778056

[B33] Fan H.-Y. Huang Y.-T. Hsieh R.-H. Chao J. C.-J. Tung Y.-C. Lee Y. L. (2018). Birthweight, Time-Varying Adiposity Growth and Early Menarche in Girls: A Mendelian Randomisation and Mediation Analysis. Obes. Res. Clin. Pract. 12, 445–451. 10.1016/j.orcp.2018.07.008 30082248

[B34] Frayling T. M. Stoneman C. E. (2018). Mendelian Randomisation in Type 2 Diabetes and Coronary Artery Disease. Curr. Opin. Genet. Development 50, 111–120. 10.1016/j.gde.2018.05.010 29935421

[B35] Gao C. Patel C. J. Michailidou K. Peters U. Gong J. Schildkraut J. (2016). Mendelian Randomization Study of Adiposity-Related Traits and Risk of Breast, Ovarian, Prostate, Lung and Colorectal Cancer. Int. J. Epidemiol. 45, 896–908. 10.1093/ije/dyw129 27427428 PMC6372135

[B36] Gharahkhani P. Ong J.-S. An J. Law M. H. Whiteman D. C. Neale R. E. (2019). Effect of Increased Body Mass index on Risk of Diagnosis or Death from Cancer. Br. J. Cancer 120, 565–570. 10.1038/s41416-019-0386-9 30733581 PMC6462026

[B37] Gianfrancesco M. A. Glymour M. M. Walter S. Rhead B. Shao X. Shen L. (2017). Causal Effect of Genetic Variants Associated with Body Mass Index on Multiple Sclerosis Susceptibility. Am. J. Epidemiol. 185, 162–171. 10.1093/aje/kww120 28073764 PMC5391720

[B38] Gill R. Stratigopoulos G. Lee J. H. Leibel R. L. (2019). Functional Genomic Characterization of the FTO Locus in African Americans. Physiol. Genomics 51, 517–528. 10.1152/physiolgenomics.00057.2019 31530225 PMC6879815

[B39] Glymour M. M. Tchetgen Tchetgen E. J. Robins J. M. (2012). Credible Mendelian Randomization Studies: Approaches for Evaluating the Instrumental Variable Assumptions. Am. J. Epidemiol. 175, 332–339. 10.1093/aje/kwr323 22247045 PMC3366596

[B40] Goodarzi M. O. (2018). Genetics of Obesity: what Genetic Association Studies Have Taught Us about the Biology of Obesity and its Complications. Lancet Diabetes Endocrinol. 6, 223–236. 10.1016/S2213-8587(17)30200-0 28919064

[B41] Greco M F. D. Minelli C. Sheehan N. A. Thompson J. R. (2015). Detecting Pleiotropy in Mendelian Randomisation Studies with Summary Data and a Continuous Outcome. Statist. Med. 34, 2926–2940. 10.1002/sim.6522 25950993

[B42] Guo J.-Z. Xiao Q. Gao S. Li X.-Q. Wu Q.-J. Gong T.-T. (2021). Review of Mendelian Randomization Studies on Ovarian Cancer. Front. Oncol. 11, 681396. 10.3389/fonc.2021.681396 34458137 PMC8385140

[B43] Guo Y. Warren Andersen S. Shu X. O. Michailidou K. Bolla M. K. Wang Q. (2016). Genetically Predicted Body Mass Index and Breast Cancer Risk: Mendelian Randomization Analyses of Data from 145,000 Women of European Descent. Plos Med. 13, e1002105. 10.1371/journal.pmed.1002105 27551723 PMC4995025

[B44] Hartwig F. P. Bowden J. Loret de Mola C. Tovo-Rodrigues L. Davey Smith G. Horta B. L. (2016). Body Mass index and Psychiatric Disorders: a Mendelian Randomization Study. Sci. Rep. 6, 32730. 10.1038/srep32730 27601421 PMC5013405

[B45] Hartwig F. P. Davey Smith G. Bowden J. (2017). Robust Inference in Summary Data Mendelian Randomization via the Zero Modal Pleiotropy assumption. Int. J. Epidemiol. 46, 1985–1998. 10.1093/ije/dyx102 29040600 PMC5837715

[B46] Hartwig F. P. Davies N. M. (2016). Why Internal Weights Should Be Avoided (Not Only) in MR-Egger Regression. Int. J. Epidemiol. 45, 1676–1678. 10.1093/ije/dyw240 27649799

[B47] Hemani G. Zheng J. Elsworth B. Wade K. H. Haberland V. Baird D. (2018b). The MR-Base Platform Supports Systematic Causal Inference across the Human Phenome. ELife 7, e34408. 10.7554/eLife.34408.001 29846171 PMC5976434

[B48] Hemani G. Bowden J. Davey Smith G. (2018a). Evaluating the Potential Role of Pleiotropy in Mendelian Randomization Studies. Hum. Mol. Genet. 27, R195–R208. 10.1093/hmg/ddy163 29771313 PMC6061876

[B49] Hertel J. K. Johansson S. Sonestedt E. Jonsson A. Lie R. T. Platou C. G. P. (2011). FTO, Type 2 Diabetes, and Weight Gain throughout Adult Life. Diabetes 60, 1637–1644. 10.2337/db10-1340 21398525 PMC3292341

[B50] Hess M. E. Hess S. Meyer K. D. Verhagen L. A. W. Koch L. Brönneke H. S. (2013). The Fat Mass and Obesity Associated Gene (Fto) Regulates Activity of the Dopaminergic Midbrain Circuitry. Nat. Neurosci. 16, 1042–1048. 10.1038/nn.3449 23817550

[B51] Howe L. D. Kanayalal R. Harrison S. Beaumont R. N. Davies A. R. Frayling T. M. (2019). Effects of Body Mass index on Relationship Status, Social Contact and Socio-Economic Position: Mendelian Randomization and Within-Sibling Study in UK Biobank. Int. J. Epidemiol. 49, 1173–1184. 10.1093/ije/dyz240 PMC7750981 31800047

[B52] Iles M. M. Law M. H. Stacey S. N. Han J. Fang S. Pfeiffer R. (2013). A Variant in FTO Shows Association with Melanoma Risk Not Due to BMI. Nat. Genet. 45, 428–432. 10.1038/ng.2571 23455637 PMC3640814

[B53] Jordan D. M. Verbanck M. Do R. (2019). HOPS: a Quantitative Score Reveals Pervasive Horizontal Pleiotropy in Human Genetic Variation Is Driven by Extreme Polygenicity of Human Traits and Diseases. Genome Biol. 20, 222. 10.1186/s13059-019-1844-7 31653226 PMC6815001

[B54] Kemp J. P. Sayers A. Smith G. D. Tobias J. H. Evans D. M. (2016). Using Mendelian Randomization to Investigate a Possible Causal Relationship between Adiposity and Increased Bone mineral Density at Different Skeletal Sites in Children. Int. J. Epidemiol. 45, 1560–1572. 10.1093/ije/dyw079 27215616 PMC5100609

[B55] Kivimäki M. Jokela M. Hamer M. Geddes J. Ebmeier K. Kumari M. (2011). Examining Overweight and Obesity as Risk Factors for Common Mental Disorders Using Fat Mass and Obesity-Associated (FTO) Genotype-Instrumented Analysis: The Whitehall II Study, 1985-2004. Am. J. Epidemiol. 173, 421–429. 10.1093/aje/kwq444 21248310 PMC3032807

[B56] Kurz C. F. Laxy M. (2020). Application of Mendelian Randomization to Investigate the Association of Body Mass Index with Health Care Costs. Med. Decis. Making 40, 156–169. 10.1177/0272989X20905809 32154779

[B57] Labrecque J. Swanson S. A. (2018). Understanding the Assumptions Underlying Instrumental Variable Analyses: a Brief Review of Falsification Strategies and Related Tools. Curr. Epidemiol. Rep. 5, 214–220. 10.1007/s40471-018-0152-1 30148040 PMC6096851

[B58] Larsson S. C. Bäck M. Rees J. M. B. Mason A. M. Burgess S. (2020). Body Mass index and Body Composition in Relation to 14 Cardiovascular Conditions in UK Biobank: a Mendelian Randomization Study. Eur. Heart J. 41, 221–226. 10.1093/eurheartj/ehz388 31195408 PMC6945523

[B59] Larsson S. C. Burgess S. Michaëlsson K. (2018). Genetic Association between Adiposity and Gout: a Mendelian Randomization Study. Rheumatology 57, 2145–2148. 10.1093/rheumatology/key229 30085130 PMC6697177

[B60] Lawlor D. A. Harbord R. M. Sterne J. A. C. Timpson N. Davey Smith G. (2008). Mendelian Randomization: Using Genes as Instruments for Making Causal Inferences in Epidemiology. Statist. Med. 27, 1133–1163. 10.1002/sim.3034 17886233

[B61] Lawlor D. A. Tilling K. Davey Smith G. (2016). Triangulation in Aetiological Epidemiology. Int. J. Epidemiol. 45, dyw314–1886. 10.1093/ije/dyw314 PMC5841843 28108528

[B62] Li H. Kilpeläinen T. O. Liu C. Zhu J. Liu Y. Hu C. (2012). Association of Genetic Variation in FTO with Risk of Obesity and Type 2 Diabetes with Data from 96,551 East and South Asians. Diabetologia 55, 981–995. 10.1007/s00125-011-2370-7 22109280 PMC3296006

[B63] Lindström S. Germain M. Germain M. Crous-Bou M. Smith E. N. Morange P.-E. (2017). Assessing the Causal Relationship between Obesity and Venous Thromboembolism through a Mendelian Randomization Study. Hum. Genet. 136, 897–902. 10.1007/s00439-017-1811-x 28528403 PMC5531049

[B64] Liu C. Mou S. Pan C. (2013). The FTO Gene Rs9939609 Polymorphism Predicts Risk of Cardiovascular Disease: a Systematic Review and Meta-Analysis. PLoS One 8, e71901. 10.1371/journal.pone.0071901 23977173 PMC3747067

[B65] Liu Q. Pan J. Berzuini C. Rutter M. K. Guo H. (2020). Integrative Analysis of Mendelian Randomization and Bayesian Colocalization Highlights Four Genes with Putative BMI-Mediated Causal Pathways to Diabetes. Sci. Rep. 10, 7476. 10.1038/s41598-020-64493-4 32366963 PMC7198550

[B66] Lor G. C. Y. Risch H. A. Fung W. T. Au Yeung S. L. Wong I. O. L. Zheng W. (2019). Reporting and Guidelines for Mendelian Randomization Analysis: A Systematic Review of Oncological Studies. Cancer Epidemiol. 62, 101577. 10.1016/j.canep.2019.101577 31377572

[B67] Lyall D. M. Celis-Morales C. Ward J. Iliodromiti S. Anderson J. J. Gill J. M. R. (2017). Association of Body Mass Index with Cardiometabolic Disease in the UK Biobank. JAMA Cardiol. 2, 882–889. 10.1001/jamacardio.2016.5804 28678979 PMC5710596

[B68] Meng X. Li X. Timofeeva M. N. He Y. Spiliopoulou A. Wei W.-Q. (2019). Phenome-wide Mendelian-Randomization Study of Genetically Determined Vitamin D on Multiple Health Outcomes Using the UK Biobank Study. Int. J. Epidemiol. 48, 1425–1434. 10.1093/ije/dyz182 31518429 PMC6857754

[B69] Minelli C. Del Greco M. F. van der Plaat D. A. Bowden J. Sheehan N. A. Thompson J. (2021). The Use of Two-Sample Methods for Mendelian Randomization Analyses on Single Large Datasets. Int. J. Epidemiol. 50, 1651–1659. 10.1093/ije/dyab084 33899104 PMC8580269

[B70] Mokry L. E. Ross S. Timpson N. J. Sawcer S. Davey Smith G. Richards J. B. (2016). Obesity and Multiple Sclerosis: A Mendelian Randomization Study. Plos Med. 13, e1002053. 10.1371/journal.pmed.1002053 27351487 PMC4924848

[B71] Morrison J. Knoblauch N. Marcus J. H. Stephens M. He X. (2020). Mendelian Randomization Accounting for Correlated and Uncorrelated Pleiotropic Effects Using Genome-wide Summary Statistics. Nat. Genet. 52, 740–747. 10.1038/s41588-020-0631-4 32451458 PMC7343608

[B72] Noyce A. J. Kia D. A. Hemani G. Nicolas A. Price T. R. De Pablo-Fernandez E. (2017). Estimating the Causal Influence of Body Mass index on Risk of Parkinson Disease: A Mendelian Randomisation Study. Plos Med. 14, e1002314. 10.1371/journal.pmed.1002314 28609445 PMC5469450

[B73] Palmer T. M. Lawlor D. A. Harbord R. M. Sheehan N. A. Tobias J. H. Timpson N. J. (2012). Using Multiple Genetic Variants as Instrumental Variables for Modifiable Risk Factors. Stat. Methods Med. Res. 21, 223–242. 10.1177/0962280210394459 21216802 PMC3917707

[B74] Pausova Z. Syme C. Abrahamowicz M. Xiao Y. Leonard G. T. Perron M. (2009). A Common Variant of the FTO Gene Is Associated with Not Only Increased Adiposity but Also Elevated Blood Pressure in French Canadians. Circ. Cardiovasc. Genet. 2, 260–269. 10.1161/CIRCGENETICS.109.857359 20031594

[B75] Pingault J.-B. Cecil C. A. M. Murray J. Munafò M. R. Viding E. (2016). Causal Inference in Psychopathology: A Systematic Review of Mendelian Randomisation Studies Aiming to Identify Environmental Risk Factors for Psychopathology. Psychopathology Rev. a4, 4–25. 10.5127/pr.038115

[B76] Qi G. Chatterjee N. (2019). Mendelian Randomization Analysis Using Mixture Models for Robust and Efficient Estimation of Causal Effects. Nat. Commun. 10, 1941. 10.1038/s41467-019-09432-2 31028273 PMC6486646

[B77] Reactive Protein Coronary Heart Disease Genetics Collaboration (Ccgc) C. Wensley F. Gao P. Burgess S. Kaptoge S. Di Angelantonio E. (2011). Association between C Reactive Protein and Coronary Heart Disease: Mendelian Randomisation Analysis Based on Individual Participant Data. BMJ 342, d548. 10.1136/bmj.d548 21325005 PMC3039696

[B78] Rees J. M. B. Wood A. M. Dudbridge F. Burgess S. (2019). Robust Methods in Mendelian Randomization via Penalization of Heterogeneous Causal Estimates. PLoS One 14, e0222362. 10.1371/journal.pone.0222362 31545794 PMC6756542

[B79] Sainani K. L. (2018). Instrumental Variables: Uses and Limitations. PM&R 10, 303–308. 10.1016/j.pmrj.2018.02.002 29551169

[B80] Sekula P. Del Greco M F. Pattaro C. Köttgen A. (2016). Mendelian Randomization as an Approach to Assess Causality Using Observational Data. Jasn 27, 3253–3265. 10.1681/ASN.2016010098 27486138 PMC5084898

[B81] Skaaby T. Taylor A. E. Thuesen B. H. Jacobsen R. K. Friedrich N. Møllehave L. T. (2018). Estimating the Causal Effect of Body Mass index on hay Fever, Asthma and Lung Function Using Mendelian Randomization. Allergy 73, 153–164. 10.1111/all.13242 28675761

[B82] Slob E. A. Groenen P. J. Thurik A. R. Rietveld C. A. (2017). A Note on the Use of Egger Regression in Mendelian Randomization Studies. Int. J. Epidemiol. 46, 2094–2097. 10.1093/ije/dyx191 29025040

[B83] Slob E. A. W. Burgess S. (2020). A Comparison of Robust Mendelian Randomization Methods Using Summary Data. Genet. Epidemiol. 44, 313–329. 10.1002/gepi.22295 32249995 PMC7317850

[B84] Sun L. Ma L. Zhang H. Cao Y. Wang C. Hou N. (2019). Fto Deficiency Reduces Anxiety- and Depression-like Behaviors in Mice via Alterations in Gut Microbiota. Theranostics 9, 721–733. 10.7150/thno.31562 30809304 PMC6376469

[B85] Sun Y.-Q. Brumpton B. M. Langhammer A. Chen Y. Kvaløy K. Mai X.-M. (2020). Adiposity and Asthma in Adults: a Bidirectional Mendelian Randomisation Analysis of the HUNT Study. Thorax 75, 202–208. 10.1136/thoraxjnl-2019-213678 31611343

[B86] Swerdlow D. I. Kuchenbaecker K. B. Shah S. Sofat R. Holmes M. V. White J. (2016). Selecting Instruments for Mendelian Randomization in the Wake of Genome-wide Association Studies. Int. J. Epidemiol. 45, 1600–1616. 10.1093/ije/dyw088 27342221 PMC5100611

[B87] Taylor A. E. Richmond R. C. Palviainen T. Loukola A. Wootton R. E. Kaprio J. (2019). The Effect of Body Mass index on Smoking Behaviour and Nicotine Metabolism: a Mendelian Randomization Study. Hum. Mol. Genet. 28, 1322–1330. 10.1093/hmg/ddy434 30561638 PMC6452214

[B88] Thompson J. R. Minelli C. Bowden J. Del Greco F. M. Gill D. Jones E. M. (2017). Mendelian Randomization Incorporating Uncertainty about Pleiotropy. Stat. Med. 36, 4627–4645. 10.1002/sim.7442 28850703

[B89] Tyrrell J. Jones S. E. Beaumont R. Astley C. M. Lovell R. Yaghootkar H. (2016). Height, Body Mass index, and Socioeconomic Status: Mendelian Randomisation Study in UK Biobank. Bmj 352, i582. 10.1136/bmj.i582 26956984 PMC4783516

[B90] Tyrrell J. Mulugeta A. Wood A. R. Zhou A. Beaumont R. N. Tuke M. A. (2019). Using Genetics to Understand the Causal Influence of Higher BMI on Depression. Int. J. Epidemiol. 48, 834–848. 10.1093/ije/dyy223 30423117 PMC6659462

[B91] van den Broek N. Treur J. L. Larsen J. K. Verhagen M. Verweij K. J. H. Vink J. M. (2018). Causal Associations between Body Mass index and Mental Health: a Mendelian Randomisation Study. J. Epidemiol. Community Health 72, 708–710. 10.1136/jech-2017-210000 29666151

[B92] VanderWeele T. J. Tchetgen Tchetgen E. J. Cornelis M. Kraft P. (2014). Methodological Challenges in Mendelian Randomization. Epidemiology 25, 427–435. 10.1097/ede.0000000000000081 24681576 PMC3981897

[B93] Vansteelandt S. Bowden J. Babanezhad M. Goetghebeur E. (2011). On Instrumental Variables Estimation of Causal Odds Ratios. Statist. Sci. 26, 403–422. 10.1214/11-STS360

[B94] Verbanck M. Chen C.-Y. Neale B. Do R. (2018). Detection of Widespread Horizontal Pleiotropy in Causal Relationships Inferred from Mendelian Randomization between Complex Traits and Diseases. Nat. Genet. 50, 693–698. 10.1038/s41588-018-0099-7 29686387 PMC6083837

[B95] Walter S. Glymour M. M. Koenen K. Liang L. Tchetgen Tchetgen E. J. Cornelis M. (2015a). Do genetic Risk Scores for Body Mass index Predict Risk of Phobic Anxiety? Evidence for a Shared Genetic Risk Factor. Psychol. Med. 45, 181–191. 10.1017/S0033291714001226 25065638 PMC4387884

[B96] Walter S. Kubzansky L. D. Koenen K. C. Liang L. Tchetgen Tchetgen E. J. Cornelis M. C. (2015b). Revisiting Mendelian Randomization Studies of the Effect of Body Mass index on Depression. Am. J. Med. Genet. 168, 108–115. 10.1002/ajmg.b.32286 PMC4387873 25656382

[B97] Wang N. Cheng J. Ning Z. Chen Y. Han B. Li Q. (2018). Type 2 Diabetes and Adiposity Induce Different Lipid Profile Disorders: A Mendelian Randomization Analysis. J. Clin. Endocrinol. Metab. 103, 2016–2025. 10.1210/jc.2017-02789 29506267

[B98] Wang T. Zhang R. Ma X. Wang S. He Z. Huang Y. (2018). Causal Association of Overall Obesity and Abdominal Obesity with Type 2 Diabetes: A Mendelian Randomization Analysis. Obesity 26, 934–942. 10.1002/oby.22167 29630776

[B99] Winter-Jensen M. Afzal S. Jess T. Nordestgaard B. G. Allin K. H. (2020). Body Mass index and Risk of Infections: a Mendelian Randomization Study of 101,447 Individuals. Eur. J. Epidemiol. 35, 347–354. 10.1007/s10654-020-00630-7 32307655

[B100] Xu L. Borges M. C. Hemani G. Lawlor D. A. (2017). The Role of Glycaemic and Lipid Risk Factors in Mediating the Effect of BMI on Coronary Heart Disease: a Two-step, Two-Sample Mendelian Randomisation Study. Diabetologia 60, 2210–2220. 10.1007/s00125-017-4396-y 28889241 PMC6342872

[B101] Xu S. Gilliland F. D. Conti D. V. (2019). Elucidation of Causal Direction between Asthma and Obesity: a Bi-directional Mendelian Randomization Study. Int. J. Epidemiol. 48, 899–907. 10.1093/ije/dyz070 31005996 PMC6659368

[B102] Zhang L. Tang L. Huang T. Fan D. (2020). Life Course Adiposity and Amyotrophic Lateral Sclerosis: A Mendelian Randomization Study. Ann. Neurol. 87, 434–441. 10.1002/ana.25671 31916305

[B103] Zhao Q. Wang J. Hemani G. Bowden J. Small D. S. (2020a). Statistical Inference in Two-Sample Summary-Data Mendelian Randomization Using Robust Adjusted Profile Score. Ann. Statist. 48, 1742–1769. 10.1214/19-AOS1866

[B104] Zhao Y. Xu Y. Wang X. Xu L. Chen J. Gao C. (2020b). Body Mass Index and Polycystic Ovary Syndrome: A 2-Sample Bidirectional Mendelian Randomization Study. J. Clin. Endocrinol. Metab. 105, 1778–1784. 10.1210/clinem/dgaa125 32163573

