# Supplementary material for: Mendelian Randomization: A Review of Methods for the Prevention, Assessment, and Discussion of Pleiotropy in Studies Using the Fat Mass and Obesity-Associated Gene as an Instrument for Adiposity
Source: Front Genet. 2022 Feb 4;13:803238. doi: 10.3389/fgene.2022.803238 (PMC8855149; doi:10.3389/fgene.2022.803238)
Supplement: Supplementary file 1 [file DataSheet1.docx]

Supplementary Material

# **Supplementary Methods**

## **Methods S1. Search strategy**

For each of the four databases listed below, the search strategy was operationalized using the keywords "Mendelian randomization", "adiposity" and their related or equivalent terms. Mendelian randomization related or equivalent terms used in this review were borrowed from the literature (Boef et al., 2015).

**PubMed**

Query:

("Mendelian Randomization Analysis"[Mesh] OR "Mendelian randomisation"[all fields] OR "Mendelian randomization"[all fields] OR (Mendelian[all fields] AND randomi*[all fields]) OR "genetic instrumental variable"[all fields] OR "genetic instrumental variables"[all fields] OR "genetic instrument"[all fields] OR "genetic instruments"[all fields] OR "genes as instruments"[all fields] OR "gene as instrument"[all fields] OR "genes as instrument"[all fields] OR "gene as instruments"[all fields] OR (instrument*[ti] AND (gene[ti] OR genes[ti] OR genetic*[ti] OR mendel*[ti])) OR (("instrumental variable"[all fields] OR "instrumental variables"[all fields] OR "instrumented analysis"[all fields] OR "instrumented analyses"[all fields] OR "instrumental variable analysis"[all fields] OR "instrumental variable analyses"[all fields] OR "instrumental variables analysis"[all fields] OR "instrumental variables analyses"[all fields]) AND (gene OR genes OR genetics OR mendel OR mendelian)) OR ("mendelian"[all fields] AND ("randomisation"[all fields] OR "randomization"[all fields] OR "randomising"[all fields] OR "randomizing"[all fields]))) AND ("adiposity"[Mesh] OR "adiposity"[all fields] OR "obesity"[all fields] OR "body mass index"[Mesh] OR "body mass index"[all fields] OR "BMI"[all fields] OR "fat mass"[all fields] OR "body fat"[all fields] OR "body fat percentage"[all fields] OR "obesity, abdominal"[Mesh] OR "waist-hip ratio"[Mesh] OR "waist-hip ratio"[all fields] OR "waist circumference"[Mesh] OR "waist circumference"[all fields])

**Medline**

Query:

(Mendelian Randomization Analysis/ OR "Mendelian randomisation".af OR "Mendelian randomization".af OR "genetic instrumental variable".af OR "genetic instrumental variables".af OR "genetic instrument".af OR "genetic instruments".af OR "mendel randomise" OR "mendel randomize" OR "mendel randomization" OR "mendel randomisation" OR "random Mendelian" OR "genes as instruments".af OR "gene as instrument".af OR "genes as instrument".af OR "gene as instruments".af) OR (instrument*.ti AND (gene.ti OR genes.ti OR genetic*.ti OR mendel*.ti)) OR (("instrumental variable".af OR "instrumental variables".af OR "instrumented analysis".af OR "instrumented analyses".af OR "instrumental variable analysis".af OR "instrumental variable analyses".af OR "instrumental variables analysis".af OR "instrumental variables analyses".af) AND (gene OR genes OR genetics OR mendel OR mendelian).af) OR ("mendelian".af AND ("randomisation".af OR "randomization".af OR "randomising".af OR "randomizing".af)) AND (adiposity/ OR "adiposity".af OR "obesity".af OR body mass index/ OR "body mass index".af OR "BMI".af OR "fat mass".af OR "body fat".af OR "body fat percentage".af OR obesity, abdominal/ OR waist-hip ratio/ OR "waist-hip ratio".af OR waist circumference/ OR "waist circumference".af)

**Embase**

Restriction: no conference abstracts.

Query:

(Mendelian Randomization Analysis/ OR "Mendelian randomisation".af OR "Mendelian randomization".af OR "genetic instrumental variable".af OR "genetic instrumental variables".af OR "genetic instrument".af OR "genetic instruments".af OR "mendel randomise" OR "mendel randomize" OR "mendel randomization" OR "mendel randomisation" OR "random Mendelian" OR "genes as instruments".af OR "gene as instrument".af OR "genes as instrument".af OR "gene as instruments".af) OR (instrument*.ti AND (gene.ti OR genes.ti OR genetic*.ti OR mendel*.ti)) OR (("instrumental variable".af OR "instrumental variables".af OR "instrumented analysis".af OR "instrumented analyses".af OR "instrumental variable analysis".af OR "instrumental variable analyses".af OR "instrumental variables analysis".af OR "instrumental variables analyses".af) AND (gene OR genes OR genetics OR mendel OR mendelian).af) OR ("mendelian".af AND ("randomisation".af OR "randomization".af OR "randomising".af OR "randomizing".af)) AND (adiposity/ OR "adiposity".af OR "obesity".af OR body mass index/ OR "body mass index".af OR "BMI".af OR "fat mass".af OR "body fat".af OR "body fat percentage".af OR obesity, abdominal/ OR waist-hip ratio/ OR "waist-hip ratio".af OR waist circumference/ OR "waist circumference".af)

**Web of Science**

Restrictions: no review, meeting abstract, editorial material, correction, letter, or book chapter.

Query:

ALL FIELDS: (("Mendelian randomisation" OR "Mendelian randomization" OR "genetic instrumental variable" OR "genetic instrumental variables" OR "genetic instrument" OR "genetic instruments" OR "mendel randomise" OR "mendel randomize" OR "mendel randomization" OR "mendel randomisation" OR "random Mendelian" OR "gene as instrument" OR "gene as instruments" OR "instrumental genetic variable" OR "instrumental genetic variables" OR "instrumental variable" OR "instrumental variable analysis") AND ("adiposity" OR "obesity" OR "body mass index" OR "BMI" OR "fat mass" OR "body fat" OR "body fat percentage" OR "abdominal obesity" OR "waist-hip ratio" OR "waist circumference"))

## **Methods S2. Specific exclusion criteria.**

Specific exclusion criteria included studies not in English, conference abstracts, letters, reports, commentaries, corrections, viewpoints, research news, perspectives, study protocols, errata, forum articles, editorials, book chapters, reviews, meta-analysis mendelian randomization (MR) studies, publications in health economics journals, theoretical or methodological papers with MR analyses examples, MR phenome-wide association studies (MR-PheWAS), MR studies with a composite exposure combining body mass index and other risk factor(s), and those involving mother-child pairs (in which the instrument and exposure are assessed in the mother and outcome in the child). In bidirectional MR studies, we only included the analysis that treated body mass index as the exposure.

# **Supplementary Tables**

**Table S1. List of the 128 studies included.**

| **Studies** | **Outcomes** |
| --- | --- |
| (Afzal et al., 2014) | Type 2 diabetes |
| (Bae and Lee, 2019) | Rheumatoid arthritis |
| (Barning and Abarin, 2016) | Physical activity |
| (Benn et al., 2016) | Any non-skin, non melanoma skin, lung, other smoking related (oral, larynx and bladder), colon, kidney, breast, prostate, and other cancers |
| (Bonnefond et al., 2017)^a^ | Plasma enzymatic activity of salivary or pancreatic amylase |
| (Brennan et al., 2009) | Lung, aero-digestive, and kidney cancers |
| (Brower et al., 2019)^a^ | Polycystic ovary syndrome |
| (Budu-Aggrey et al., 2019)^a^ | Psoriasis |
| (Busch et al., 2019) | Timing of puberty |
| (Carreras-Torres et al., 2016) | Different histological types of lung cancer |
| (Carreras-Torres et al., 2018) | Smoking behaviour |
| (Censin et al., 2017) | Type 1 diabetes |
| (Censin et al., 2019) | Several non-communicable diseases and cardiometabolic risk factors |
| (Chatterjee et al., 2017) | Atrial fibrillation |
| (Chen, Fan, Huang, et al., 2019)^a^ | Asthma |
| (Chen, Fan, Yang, et al., 2019)^a^ | Early puberty |
| (Colak et al., 2016) | Wheezing and asthma |
| (Cole et al., 2016) | Coronary artery disease |
| (Corbin et al., 2016) | Type 2 diabetes |
| (Dale et al., 2017) | Coronary artery disease, type 2 diabetes mellitus, and major stroke subtypes (primary outcomes), and 18 cardiometabolic traits (secondary outcomes) |
| (Davies et al., 2015) | Prostate cancer risk and mortality |
| (den Hollander et al., 2017)^a^ | Helicobacter pylori colonization |
| (Dixon et al., 2016) | Subtypes of ovarian cancer |
| (Dusingize et al., 2020) | Melanoma |
| (Eriksson et al., 2017)^a^ | Serum testosterone |
| (Fall et al., 2013) | 24 cardiometabolic phenotypes |
| (Fall et al., 2015) | 14 cardiovascular risk factors |
| (Fan et al., 2018) | Early age at menarche |
| (Fussey et al., 2020) | Benign nodular thyroid disease and differentiated thyroid cancer |
| (Gao et al., 2016) | Breast, ovarian, prostate, colorectal, and lung cancers |
| (Geng et al., 2018) | Type 2 diabetes, coronary artery disease, and chronic kidney disease (primary outcomes); several cardiometabolic traits (secondary outcomes) |
| (Gharahkhani et al., 2019) | Overall cancer risk and mortality, and specific type cancer risk |
| (Gianfrancesco, Glymour, et al., 2017) | Multiple sclerosis susceptibility |
| (Gianfrancesco, Stridh, et al., 2017) | Pediatric-onset multiple sclerosis |
| (Granell et al., 2014) | Current asthma and asthma subtypes |
| (Guo et al., 2017) | Breast cancer survival |
| (Guo et al., 2016) | Breast cancer |
| (Hagg et al., 2015) | Coronary heart disease, heart failure, and ischaemic stroke |
| (Hartwig et al., 2016) | Bipolar and major depressive disorders, and schizophrenia |
| (Holmes et al., 2014) | Several cardiometabolic traits and events |
| (Howe et al., 2019) | Six measures of socio-economic position and three measures of social contact |
| (Howe et al., 2017) | Body dissatisfaction and smoking behaviour |
| (Huang et al., 2016) | Peripheral arterial disease |
| (Hung et al., 2014) | Major depression |
| (Jacobs et al., 2020)^a^ | Multiple sclerosis |
| (Jarvis et al., 2016) | Colorectal cancer |
| (Jokela et al., 2012) | Depressive symptoms |
| (Kaltoft et al., 2020) | Aortic valve stenosis and replacement |
| (Kemp et al., 2016)^a^ | Bone mineral density |
| (Kivimäki et al., 2011) | Common mental disorders |
| (Kivimaki et al., 2008) | Adult carotid intima-media thickness and various atherosclerotic risk factors |
| (Klovaite et al., 2015) | First events of deep venous thrombosis with or without pulmonary embolism |
| (Kurz and Laxy, 2020) | Total annual health care costs |
| (Larsson et al., 2020) | 14 cardiovascular conditions |
| (Larsson et al., 2018) | Gout risk and serum urate concentrations |
| (Lawlor et al., 2011) | Psychological distress |
| (Lee et al., 2018) | Hypertension |
| (Lee et al., 2019) | Bone health measures |
| (Lewis et al., 2010) | Prostate cancer |
| (Lim et al., 2009) | Cataract outcomes |
| (Lindstrom et al., 2017) | Venous thromboembolism |
| (Liu et al., 2020) | Diabetes |
| (Lyall et al., 2017) | Stroke, coronary heart disease, hypertension, type 2 diabetes, systolic and diastolic blood pressure, and pulse rate. |
| (Lyngdoh et al., 2012)^a^ | Serum uric acid |
| (Mao et al., 2017) | Gastric cancer |
| (Marini et al., 2020)^a^ | Multiple cerebrovascular disease phenotypes |
| (Martins-Silva et al., 2019)^a^ | Attention-deficit hyperactivity disorder |
| (Mokry et al., 2016) | Multiple sclerosis |
| (Mukherjee et al., 2015) | Alzheimer’s disease and dementia |
| (Mumby et al., 2011) | Early menarche |
| (Nordestgaard et al., 2012) | Ischemic heart disease |
| (Nordestgaard et al., 2017) | Alzheimer’s disease |
| (Noyce et al., 2017) | Parkinson disease |
| (Ooi et al., 2019)^a^ | Breast size and breast cancer risk |
| (Painter et al., 2016) | Endometrial cancer |
| (Panoutsopoulou et al., 2014) | Knee and hip osteoarthritis |
| (Qian, Rookus, et al., 2019) | Ovarian cancer risk |
| (Qian, Wang, et al., 2019) | Breast cancer risk |
| (Reed et al., 2017)^a^ | Disordered eating patterns |
| (Reed et al., 2020)^a^ | DNA methylation |
| (Richardson et al., 2020) | Coronary artery disease, type 2 diabetes, breast cancer, and prostate cancer |
| (Richmond et al., 2014)^a^ | Physical activity level |
| (Robinson et al., 2020) | Postoperative complications |
| (Rode et al., 2014) | Short telomere length |
| (Schnurr et al., 2018) | Sedentary time and physical activity |
| (Shu et al., 2019) | Breast cancer risk |
| (Shungin et al., 2015) | Periodontitis |
| (Skaaby et al., 2018) | Asthma, hay fever, allergic sensitization, serum total immunoglobulin E, forced expiratory volume in one-second and forced vital capacity |
| (Speed et al., 2019)^a^ | Depression |
| (Stender et al., 2013) | Symptomatic gallstone disease |
| (Sun et al., 2020)^a^ | Asthma |
| (Sun et al., 2019) | All cause and cause specific (cardiovascular, cancer, and non-cardiovascular non-cancer) and incident diseases (cardiovascular and cancer) |
| (Takahashi et al., 2019) | Meningioma |
| (Tan et al., 2019) | Cataract |
| (Taylor et al., 2019)^a^ | Smoking behaviours, DNA methylation, cotinine levels, and nicotine metabolite ratio |
| (Taylor et al., 2016) | Free T_3_ and T_4_ levels |
| (Thakkinstian et al., 2014)^a^ | Circulating fetuin-A level |
| (Thrift et al., 2015) | Colorectal cancer |
| (Thrift et al., 2014) | Esophageal adenocarcinoma and Barrett’s esophagus |
| (Timpson et al., 2009) | Systolic and diastolic blood pressure |
| (Timpson et al., 2011)^a^ | Circulating C-reactive protein |
| (Todd et al., 2015) | Diabetic kidney disease |
| (Tyrrell et al., 2016) | Age completed full time education, degree level education, job class, annual household income, and Townsend deprivation index. |
| (Tyrrell et al., 2019) | Depression |
| (van den Broek et al., 2018)^a^ | Subjective well-being and depressive symptoms |
| (van 't Hof et al., 2017) | Intracranial and abdominal aortic aneurysms |
| (Varbo et al., 2015) | Ischemic heart disease |
| (Vasan et al., 2019) | Circulating plasma succinate and 12,13-dihydroxy-9Z-octadecenoic acid |
| (Vimaleswaran et al., 2013)^a^ | 25-hydroxyvitamin D |
| (Wade, Carslake, et al., 2018) | All-cause and cause-specific mortality |
| (Wade, Chiesa, et al., 2018) | Blood pressure, heart rate, carotid femoral pulse wave velocity, carotid intima-media thickness, and left ventricular mass index |
| (Wainberg et al., 2019) | Type 2 diabetes |
| (Walter, Glymour, et al., 2015) | Phobic anxiety |
| (Walter, Kubzansky, et al., 2015) | Depression |
| (Wang, Cheng, et al., 2018) | Total cholesterol, high-density lipoprotein cholesterol, low-density lipoprotein cholesterol, and triglycerides |
| (Wang, Lu, et al., 2018) | Blood lead level |
| (Wang et al., 2016) | Insulin secretion and sensitivity |
| (Wang, Zhang, et al., 2018) | Type 2 diabetes development, insulin secretion, and insulin resistant traits |
| (Warodomwichit et al., 2013) | Bone mineral density |
| (Welsh et al., 2010)^a^ | Circulating levels of C-reactive protein and leptin |
| (Winter-Jensen et al., 2020) | Hospital contacts related to infections |
| (Wurtz et al., 2014) | 82 metabolic measures |
| (Xu et al., 2017)^a^ | Coronary heart disease |
| (Xu et al., 2019)^a^ | Asthma |
| (Zeng et al., 2019)^a^ | Amyotrophic lateral sclerosis |
| (Zhang et al., 2020) | Amyotrophic lateral sclerosis |
| (Zhao, Xu, et al., 2020)^a^ | Polycystic ovary syndrome |
| (Zhou et al., 2019) | Alzheimer’s disease |
| ^a^ Bidirectional mendelian randomization study. We only analysed the analysis that treated body mass index as exposure. | |

**Table S2. Frequency of use of 31 *FTO* single-nucleotide polymorphisms as instruments for body mass index.**

| ***FTO* SNPs** | **n/128** | **References** |
| --- | --- | --- |
| rs1558902 | 64 | (Bae and Lee, 2019; Bonnefond et al., 2017; Budu-Aggrey et al., 2019; Busch et al., 2019; Carreras-Torres et al., 2016; Chatterjee et al., 2017; Cole et al., 2016; Corbin et al., 2016; Dale et al., 2017; Davies et al., 2015; Dixon et al., 2016; Dusingize et al., 2020; Eriksson et al., 2017; Fall et al., 2015; Fussey et al., 2020; Gao et al., 2016; Gianfrancesco, Glymour, et al., 2017; Gianfrancesco, Stridh, et al., 2017; Granell et al., 2014; Guo et al., 2017; Guo et al., 2016; Hartwig et al., 2016; Howe et al., 2019; Howe et al., 2017; Jarvis et al., 2016; Jokela et al., 2012; Kurz and Laxy, 2020; Larsson et al., 2020; Larsson et al., 2018; Lindstrom et al., 2017; Lyall et al., 2017; Mao et al., 2017; Mokry et al., 2016; Mukherjee et al., 2015; Noyce et al., 2017; Ooi et al., 2019; Painter et al., 2016; Qian, Rookus, et al., 2019; Qian, Wang, et al., 2019; Reed et al., 2017; Reed et al., 2020; Richmond et al., 2014; Robinson et al., 2020; Shu et al., 2019; Sun et al., 2020; Sun et al., 2019; Taylor et al., 2019; Taylor et al., 2016; Thrift et al., 2015; Todd et al., 2015; Tyrrell et al., 2016; Tyrrell et al., 2019; van 't Hof et al., 2017; Wade, Carslake, et al., 2018; Wade, Chiesa, et al., 2018; Wainberg et al., 2019; Walter, Glymour, et al., 2015; Walter, Kubzansky, et al., 2015; Wang et al., 2016; Wang, Zhang, et al., 2018; Wurtz et al., 2014; Xu et al., 2017; Xu et al., 2019; Zhang et al., 2020) |
| rs9939609 | 28 | (Afzal et al., 2014; Barning and Abarin, 2016; Benn et al., 2016; Brennan et al., 2009; Chen, Fan, Yang, et al., 2019; Colak et al., 2016; Fall et al., 2013; Kaltoft et al., 2020; Kivimaki et al., 2008; Klovaite et al., 2015; Lawlor et al., 2011; Lee et al., 2018; Lewis et al., 2010; Lim et al., 2009; Nordestgaard et al., 2012; Nordestgaard et al., 2017; Rode et al., 2014; Skaaby et al., 2018; Stender et al., 2013; Tan et al., 2019; Thakkinstian et al., 2014; Timpson et al., 2009; Timpson et al., 2011; Varbo et al., 2015; Vimaleswaran et al., 2013; Warodomwichit et al., 2013; Welsh et al., 2010; Winter-Jensen et al., 2020) |
| rs1421085 | 20 | (Brower et al., 2019; Carreras-Torres et al., 2018; Censin et al., 2017; den Hollander et al., 2017; Dusingize et al., 2020; Gao et al., 2016; Geng et al., 2018; Hagg et al., 2015; Holmes et al., 2014; Jacobs et al., 2020; Jarvis et al., 2016; Kemp et al., 2016; Kivimäki et al., 2011; Lim et al., 2009; Liu et al., 2020; Martins-Silva et al., 2019; Schnurr et al., 2018; van den Broek et al., 2018; Zhang et al., 2020; Zhou et al., 2019) |
| rs1121980 | 5 | (Barning and Abarin, 2016; Lyngdoh et al., 2012; Mumby et al., 2011; Shungin et al., 2015; Skaaby et al., 2018) |
| rs17817449 | 5 | (Huang et al., 2016; Lee et al., 2019; Lim et al., 2009; Wang, Cheng, et al., 2018; Wang, Lu, et al., 2018) |
| rs7193144 | 3 | (Censin et al., 2019; Lim et al., 2009; Lyngdoh et al., 2012) |
| rs11644943 | 3 | (Chen, Fan, Huang, et al., 2019; Chen, Fan, Yang, et al., 2019; Fan et al., 2018) |
| rs11642015 | 3 | (Gharahkhani et al., 2019; Speed et al., 2019; Zhao, Xu, et al., 2020) |
| rs9930506 | 2 | (den Hollander et al., 2017; Thrift et al., 2014) |
| rs2075205 | 2 | (Gharahkhani et al., 2019; Vasan et al., 2019) |
| rs8050136 | 1 | (Lim et al., 2009) |
| rs3751812 | 1 | (Hung et al., 2014) |
| rs8044769 | 1 | (Panoutsopoulou et al., 2014) |
| rs62048402 | 1 | (Fan et al., 2018) |
| rs9939973 | 1 | (Lim et al., 2009) |
| rs9940128 | 1 | (Lim et al., 2009) |
| rs1121980 | 1 | (Lim et al., 2009) |
| rs9926289 | 1 | (Lim et al., 2009) |
| rs1075440 | 1 | (Marini et al., 2020) |
| rs1421090 | 1 | (Marini et al., 2020) |
| rs56094641 | 1 | (Richardson et al., 2020) |
| rs2072518 | 1 | (Takahashi et al., 2019) |
| rs8047395 | 1 | (Takahashi et al., 2019) |
| rs12597712 | 1 | (Zeng et al., 2019) |
| rs9931164 | 1 | (Jacobs et al., 2020) |
| rs3751813 | 1 | (Vasan et al., 2019) |
| rs2540769 | 1 | (Lyngdoh et al., 2012) |
| rs2665272 | 1 | (Lyngdoh et al., 2012) |
| rs6499658 | 1 | (Lyngdoh et al., 2012) |
| rs8060649 | 1 | (Speed et al., 2019) |
| rs16952465 | 1 | (Gharahkhani et al., 2019) |
| **Abbreviations:** *FTO*, fat mass and obesity*-*associated; SNPs, single-nucleotide polymorphisms. | | |

**Table S3. Characteristics of included studies.**

| **Characteristics of studies** | **n** | **References** |
| --- | --- | --- |
| **Types of data used** | **n=128** |  |
| One-sample only | 82 | (Afzal et al., 2014; Barning and Abarin, 2016; Benn et al., 2016; Bonnefond et al., 2017; Brennan et al., 2009; Chatterjee et al., 2017; Chen, Fan, Huang, et al., 2019; Colak et al., 2016; Cole et al., 2016; Dale et al., 2017; Davies et al., 2015; den Hollander et al., 2017; Dixon et al., 2016; Eriksson et al., 2017; Fall et al., 2013; Fall et al., 2015; Fan et al., 2018; Gharahkhani et al., 2019; Gianfrancesco, Glymour, et al., 2017; Gianfrancesco, Stridh, et al., 2017; Granell et al., 2014; Guo et al., 2017; Hagg et al., 2015; Holmes et al., 2014; Howe et al., 2017; Huang et al., 2016; Hung et al., 2014; Jarvis et al., 2016; Jokela et al., 2012; Kaltoft et al., 2020; Kemp et al., 2016; Kivimäki et al., 2011; Kivimaki et al., 2008; Klovaite et al., 2015; Larsson et al., 2020; Lawlor et al., 2011; Lee et al., 2018; Lee et al., 2019; Lewis et al., 2010; Lim et al., 2009; Lyall et al., 2017; Lyngdoh et al., 2012; Mao et al., 2017; Mukherjee et al., 2015; Mumby et al., 2011; Nordestgaard et al., 2012; Painter et al., 2016; Panoutsopoulou et al., 2014; Qian, Rookus, et al., 2019; Qian, Wang, et al., 2019; Reed et al., 2020; Richmond et al., 2014; Robinson et al., 2020; Rode et al., 2014; Schnurr et al., 2018; Shungin et al., 2015; Stender et al., 2013; Sun et al., 2020; Sun et al., 2019; Tan et al., 2019; Taylor et al., 2016; Thakkinstian et al., 2014; Thrift et al., 2015; Thrift et al., 2014; Timpson et al., 2009; Timpson et al., 2011; Todd et al., 2015; Tyrrell et al., 2016; van 't Hof et al., 2017; Varbo et al., 2015; Vimaleswaran et al., 2013; Wainberg et al., 2019; Walter, Glymour, et al., 2015; Walter, Kubzansky, et al., 2015; Wang, Cheng, et al., 2018; Wang, Lu, et al., 2018; Wang et al., 2016; Wang, Zhang, et al., 2018; Warodomwichit et al., 2013; Welsh et al., 2010; Winter-Jensen et al., 2020; Wurtz et al., 2014) |
| Two-sample only | 30 | (Bae and Lee, 2019; Brower et al., 2019; Busch et al., 2019; Carreras-Torres et al., 2016; Censin et al., 2017; Corbin et al., 2016; Dusingize et al., 2020; Gao et al., 2016; Geng et al., 2018; Hartwig et al., 2016; Jacobs et al., 2020; Larsson et al., 2018; Lindstrom et al., 2017; Liu et al., 2020; Marini et al., 2020; Martins-Silva et al., 2019; Mokry et al., 2016; Noyce et al., 2017; Ooi et al., 2019; Richardson et al., 2020; Speed et al., 2019; Takahashi et al., 2019; Taylor et al., 2019; van den Broek et al., 2018; Vasan et al., 2019; Xu et al., 2017; Zeng et al., 2019; Zhang et al., 2020; Zhao, Xu, et al., 2020; Zhou et al., 2019) |
| Both one and two samples | 16 | (Budu-Aggrey et al., 2019; Carreras-Torres et al., 2018; Censin et al., 2019; Chen, Fan, Yang, et al., 2019; Fussey et al., 2020; Guo et al., 2016; Howe et al., 2019; Kurz and Laxy, 2020; Nordestgaard et al., 2017; Reed et al., 2017; Shu et al., 2019; Skaaby et al., 2018; Tyrrell et al., 2019; Wade, Carslake, et al., 2018; Wade, Chiesa, et al., 2018; Xu et al., 2019) |
| **Types of instruments used in the main analysis** | **n=128** |  |
| GRS only | 63 | (Afzal et al., 2014; Benn et al., 2016; Carreras-Torres et al., 2018; Chen, Fan, Huang, et al., 2019; Chen, Fan, Yang, et al., 2019; Colak et al., 2016; Cole et al., 2016; Davies et al., 2015; den Hollander et al., 2017; Dixon et al., 2016; Eriksson et al., 2017; Fall et al., 2015; Fan et al., 2018; Gianfrancesco, Glymour, et al., 2017; Gianfrancesco, Stridh, et al., 2017; Granell et al., 2014; Guo et al., 2017; Guo et al., 2016; Hagg et al., 2015; Holmes et al., 2014; Howe et al., 2019; Howe et al., 2017; Huang et al., 2016; Jokela et al., 2012; Kaltoft et al., 2020; Lee et al., 2018; Lee et al., 2019; Lyall et al., 2017; Lyngdoh et al., 2012; Mao et al., 2017; Mukherjee et al., 2015; Mumby et al., 2011; Nordestgaard et al., 2012; Nordestgaard et al., 2017; Painter et al., 2016; Qian, Rookus, et al., 2019; Qian, Wang, et al., 2019; Reed et al., 2020; Richmond et al., 2014; Robinson et al., 2020; Rode et al., 2014; Schnurr et al., 2018; Shungin et al., 2015; Skaaby et al., 2018; Stender et al., 2013; Sun et al., 2020; Sun et al., 2019; Taylor et al., 2016; Thrift et al., 2015; Thrift et al., 2014; Todd et al., 2015; Tyrrell et al., 2016; Varbo et al., 2015; Vimaleswaran et al., 2013; Wade, Carslake, et al., 2018; Wade, Chiesa, et al., 2018; Wang, Cheng, et al., 2018; Wang, Lu, et al., 2018; Wang et al., 2016; Wang, Zhang, et al., 2018; Welsh et al., 2010; Winter-Jensen et al., 2020; Wurtz et al., 2014) |
| Multiple IVs only | 40 | (Bae and Lee, 2019; Bonnefond et al., 2017; Brower et al., 2019; Budu-Aggrey et al., 2019; Busch et al., 2019; Carreras-Torres et al., 2016; Censin et al., 2017; Corbin et al., 2016; Dale et al., 2017; Dusingize et al., 2020; Gao et al., 2016; Geng et al., 2018; Gharahkhani et al., 2019; Hartwig et al., 2016; Jacobs et al., 2020; Jarvis et al., 2016; Larsson et al., 2020; Larsson et al., 2018; Lindstrom et al., 2017; Liu et al., 2020; Marini et al., 2020; Martins-Silva et al., 2019; Mokry et al., 2016; Noyce et al., 2017; Ooi et al., 2019; Richardson et al., 2020; Speed et al., 2019; Takahashi et al., 2019; Taylor et al., 2019; Timpson et al., 2009; van 't Hof et al., 2017; van den Broek et al., 2018; Vasan et al., 2019; Wainberg et al., 2019; Xu et al., 2017; Xu et al., 2019; Zeng et al., 2019; Zhang et al., 2020; Zhao, Xu, et al., 2020; Zhou et al., 2019) |
| Single IVs only | 13 | (Barning and Abarin, 2016; Brennan et al., 2009; Fall et al., 2013; Kivimäki et al., 2011; Kivimaki et al., 2008; Klovaite et al., 2015; Lewis et al., 2010; Lim et al., 2009; Panoutsopoulou et al., 2014; Tan et al., 2019; Thakkinstian et al., 2014; Timpson et al., 2011; Warodomwichit et al., 2013) |
| Both GRS and multiple IVs | 6 | (Censin et al., 2019; Fussey et al., 2020; Kurz and Laxy, 2020; Reed et al., 2017; Shu et al., 2019; Tyrrell et al., 2019) |
| Both GRS and single IVs | 5 | (Chatterjee et al., 2017; Hung et al., 2014; Kemp et al., 2016; Walter, Glymour, et al., 2015; Walter, Kubzansky, et al., 2015) |
| Both single and multiple IVs | 1 | (Lawlor et al., 2011) |
| **Types of GRS used among studies using GRS as IV in the main analysis** | **74** |  |
| Weighted GRS only | 48 | (Carreras-Torres et al., 2018; Censin et al., 2019; Chatterjee et al., 2017; Chen, Fan, Huang, et al., 2019; Chen, Fan, Yang, et al., 2019; Davies et al., 2015; Dixon et al., 2016; Eriksson et al., 2017; Fan et al., 2018; Fussey et al., 2020; Gianfrancesco, Glymour, et al., 2017; Granell et al., 2014; Guo et al., 2017; Guo et al., 2016; Holmes et al., 2014; Howe et al., 2019; Howe et al., 2017; Huang et al., 2016; Hung et al., 2014; Lee et al., 2019; Lyall et al., 2017; Mao et al., 2017; Mukherjee et al., 2015; Painter et al., 2016; Qian, Rookus, et al., 2019; Qian, Wang, et al., 2019; Reed et al., 2017; Reed et al., 2020; Richmond et al., 2014; Robinson et al., 2020; Schnurr et al., 2018; Shu et al., 2019; Sun et al., 2020; Sun et al., 2019; Taylor et al., 2016; Thrift et al., 2015; Todd et al., 2015; Tyrrell et al., 2016; Tyrrell et al., 2019; Vimaleswaran et al., 2013; Wade, Carslake, et al., 2018; Wade, Chiesa, et al., 2018; Walter, Glymour, et al., 2015; Walter, Kubzansky, et al., 2015; Wang, Cheng, et al., 2018; Wang, Lu, et al., 2018; Wang, Zhang, et al., 2018; Wurtz et al., 2014) |
| Unweighted GRS only | 19 | (Afzal et al., 2014; Benn et al., 2016; Colak et al., 2016; Cole et al., 2016; den Hollander et al., 2017; Fall et al., 2015; Hagg et al., 2015; Jokela et al., 2012; Kemp et al., 2016; Kurz and Laxy, 2020; Lyngdoh et al., 2012; Mumby et al., 2011; Nordestgaard et al., 2012; Rode et al., 2014; Shungin et al., 2015; Skaaby et al., 2018; Stender et al., 2013; Varbo et al., 2015; Welsh et al., 2010) |
| Both weighted and unweighted GRS | 7 | (Gianfrancesco, Stridh, et al., 2017; Kaltoft et al., 2020; Lee et al., 2018; Nordestgaard et al., 2017; Thrift et al., 2014; Wang et al., 2016; Winter-Jensen et al., 2020) |
| **Abbreviations:** GRS, genetic risk score; IVs, instrumental variables; SNPs, single*-*nucleotide polymorphisms; *FTO*, fat mass and obesity-associated. | | |

**Table S4. Approaches cited for preventing pleiotropy in the selection of IVs.**

| **Approaches** | **n/33** | **References** |
| --- | --- | --- |
| Exclude selected SNPs known or suspected to be pleiotropic | 12 | (Bonnefond et al., 2017; Censin et al., 2017; Chen, Fan, Huang, et al., 2019; Chen, Fan, Yang, et al., 2019; Fan et al., 2018; Howe et al., 2019; Sun et al., 2020; Sun et al., 2019; Tyrrell et al., 2016; Tyrrell et al., 2019; Xu et al., 2019; Zhou et al., 2019) |
| Use of multiple (independent) SNPs combined in a GRS as IVs | 10 | (Benn et al., 2016; Fall et al., 2015; Holmes et al., 2014; Kurz and Laxy, 2020; Shungin et al., 2015; Taylor et al., 2016; Todd et al., 2015; Varbo et al., 2015; Vimaleswaran et al., 2013; Winter-Jensen et al., 2020) |
| Use of multiple (independent) SNPs as IVs | 10^1^ | (Carreras-Torres et al., 2016; Colak et al., 2016; Dale et al., 2017; Dixon et al., 2016; Eriksson et al., 2017; Lawlor et al., 2011; Lewis et al., 2010; Nordestgaard et al., 2012; Stender et al., 2013; Wurtz et al., 2014) |
| Use a single SNP as IV | 1 | (Tan et al., 2019) |
| **Abbreviations:** GRS, genetic risk score; SNP, single*-*nucleotide polymorphisms; IV, instrumental variable.  ^1^These 10 articles discussed the use of multiple independent SNPs without explicitly mentioning GRS even if six (Colak et al., 2016; Dixon et al., 2016; Eriksson et al., 2017; Nordestgaard et al., 2012; Stender et al., 2013; Wurtz et al., 2014) of the 10 in fact used a GRS as IV in the main analysis. | | |

**Table S5. Methods cited for detection or accounting the violation of the independence and/or restriction exclusion assumptions.**

| **Methods^1^** | **n/128** | **Studies specifically referred to the method for ‘pleiotropy’ assessment / control / robustness (n=95)** | | **Studies not specifically referred to the method for ‘pleiotropy’ assessment / control / robustness (n=96)** | |
| --- | --- | --- | --- | --- | --- |
|  |  | **n** | **References** | **n** | **References** |
| **Robust and other IV estimation methods^2^** | **70** | **69** |  | **23** |  |
| Robust methods | 69 | 68 |  | 20 |  |
| MR-Egger | 69**^3^** | 68 | (Bae and Lee, 2019; Bonnefond et al., 2017; Brower et al., 2019; Budu-Aggrey et al., 2019; Busch et al., 2019; Carreras-Torres et al., 2016; Carreras-Torres et al., 2018; Censin et al., 2017; Censin et al., 2019; Chen, Fan, Huang, et al., 2019; Chen, Fan, Yang, et al., 2019; Corbin et al., 2016; Dale et al., 2017; Dixon et al., 2016; Dusingize et al., 2020; Fussey et al., 2020; Gao et al., 2016; Geng et al., 2018; Gharahkhani et al., 2019; Guo et al., 2017; Guo et al., 2016; Hartwig et al., 2016; Howe et al., 2019; Jacobs et al., 2020; Jarvis et al., 2016; Kemp et al., 2016; Kurz and Laxy, 2020; Larsson et al., 2020; Larsson et al., 2018; Lee et al., 2019; Lindstrom et al., 2017; Liu et al., 2020; Lyall et al., 2017; Mao et al., 2017; Marini et al., 2020; Martins-Silva et al., 2019; Mokry et al., 2016; Noyce et al., 2017; Ooi et al., 2019; Painter et al., 2016; Qian, Rookus, et al., 2019; Qian, Wang, et al., 2019; Reed et al., 2017; Richardson et al., 2020; Schnurr et al., 2018; Shu et al., 2019; Skaaby et al., 2018; Speed et al., 2019; Sun et al., 2020; Sun et al., 2019; Takahashi et al., 2019; Taylor et al., 2019; Tyrrell et al., 2016; Tyrrell et al., 2019; van 't Hof et al., 2017; van den Broek et al., 2018; Vasan et al., 2019; Wade, Carslake, et al., 2018; Wade, Chiesa, et al., 2018; Wainberg et al., 2019; Wang, Zhang, et al., 2018; Winter-Jensen et al., 2020; Xu et al., 2017; Xu et al., 2019; Zeng et al., 2019; Zhang et al., 2020; Zhao, Xu, et al., 2020; Zhou et al., 2019) | 1 | (Fan et al., 2018) |
| Median-based methods | 44 | 26 | (Bae and Lee, 2019; Budu-Aggrey et al., 2019; Busch et al., 2019; Carreras-Torres et al., 2016; Carreras-Torres et al., 2018; Censin et al., 2017; Censin et al., 2019; Chen, Fan, Huang, et al., 2019; Chen, Fan, Yang, et al., 2019; Dale et al., 2017; Fussey et al., 2020; Geng et al., 2018; Hartwig et al., 2016; Howe et al., 2019; Marini et al., 2020; Martins-Silva et al., 2019; Mokry et al., 2016; Reed et al., 2017; Skaaby et al., 2018; Speed et al., 2019; Taylor et al., 2019; Tyrrell et al., 2019; van den Broek et al., 2018; Vasan et al., 2019; Wade, Chiesa, et al., 2018; Xu et al., 2019) | 18 | (Corbin et al., 2016; Dusingize et al., 2020; Gharahkhani et al., 2019; Jacobs et al., 2020; Kurz and Laxy, 2020; Larsson et al., 2020; Larsson et al., 2018; Liu et al., 2020; Ooi et al., 2019; Sun et al., 2019; van 't Hof et al., 2017; Wade, Carslake, et al., 2018; Wang, Zhang, et al., 2018; Xu et al., 2017; Zeng et al., 2019; Zhang et al., 2020; Zhao, Xu, et al., 2020; Zhou et al., 2019) |
| Mode-based methods | 9 | 5 | (Budu-Aggrey et al., 2019; Carreras-Torres et al., 2018; Martins-Silva et al., 2019; Taylor et al., 2019; Zhou et al., 2019) | 4 | (Jacobs et al., 2020; Marini et al., 2020; Ooi et al., 2019; Wade, Carslake, et al., 2018) |
| MR-PRESSO | 6 | 5 | (Marini et al., 2020; Ooi et al., 2019; Xu et al., 2019; Zeng et al., 2019; Zhou et al., 2019) | 1 | (Larsson et al., 2020) |
| MR-RAPS | 1 | 0 |  | 1 | (Liu et al., 2020) |
| Other IV estimation methods | 13 | 6 |  | 8 |  |
| Multivariable MR | 6 | 3 | (Censin et al., 2017; Huang et al., 2016; Kemp et al., 2016) | 3 | (Dale et al., 2017; Jacobs et al., 2020; Marini et al., 2020) |
| IVW methods | 4 | 3 | (Chen, Fan, Yang, et al., 2019; Howe et al., 2019; Jarvis et al., 2016) | 1 | (Fan et al., 2018) |
| Likelihood-based methods | 4 | 0 |  | 4 | (Brower et al., 2019; Censin et al., 2017; Gharahkhani et al., 2019; Jacobs et al., 2020) |
| **Methods to detect heterogeneity of estimated causal effects across IVs** | **72** | **46** |  | **46** |  |
| Graphical assessment: scatter plots, forest plots, funnel plots, leave-one-out plots, and histogram | 34 | 20 | (Bae and Lee, 2019; Censin et al., 2017; Chen, Fan, Huang, et al., 2019; Corbin et al., 2016; Dusingize et al., 2020; Fan et al., 2018; Gharahkhani et al., 2019; Granell et al., 2014; Hagg et al., 2015; Hartwig et al., 2016; Kemp et al., 2016; Lyall et al., 2017; Mokry et al., 2016; Noyce et al., 2017; Painter et al., 2016; Sun et al., 2019; Wade, Carslake, et al., 2018; Winter-Jensen et al., 2020; Zhao, Xu, et al., 2020; Zhou et al., 2019) | 19 | (Brower et al., 2019; Busch et al., 2019; Carreras-Torres et al., 2018; Chen, Fan, Huang, et al., 2019; Corbin et al., 2016; Davies et al., 2015; Dusingize et al., 2020; Gao et al., 2016; Guo et al., 2017; Guo et al., 2016; Larsson et al., 2018; Liu et al., 2020; Noyce et al., 2017; Ooi et al., 2019; Qian, Wang, et al., 2019; Takahashi et al., 2019; Taylor et al., 2019; Zeng et al., 2019; Zhou et al., 2019) |
| Statistical criteria and tests: I^2^, r^2^, H statistic, Cochran's Q, Rucker's Q, over-identification tests^4^ | 30 | 7 | (Censin et al., 2017; Granell et al., 2014; Hartwig et al., 2016; Kurz and Laxy, 2020; Painter et al., 2016; Qian, Rookus, et al., 2019; Reed et al., 2017) | 23 | (Bae and Lee, 2019; Carreras-Torres et al., 2018; Chen, Fan, Huang, et al., 2019; Dusingize et al., 2020; Fan et al., 2018; Gianfrancesco, Glymour, et al., 2017; Holmes et al., 2014; Larsson et al., 2018; Lawlor et al., 2011; Mao et al., 2017; Marini et al., 2020; Mukherjee et al., 2015; Nordestgaard et al., 2012; Noyce et al., 2017; Qian, Wang, et al., 2019; Richardson et al., 2020; Sun et al., 2020; Tyrrell et al., 2019; van den Broek et al., 2018; Walter, Glymour, et al., 2015; Walter, Kubzansky, et al., 2015; Zeng et al., 2019; Zhou et al., 2019) |
| Comparisons of estimated MR causal effects across IVs (GRS or multiple IVs) | 39 | 25 |  | 14 |  |
| Before and after exclusion of SNPs suspected of pleiotropy | 33 | 20 | (Benn et al., 2016; Carreras-Torres et al., 2016; Carreras-Torres et al., 2018; Davies et al., 2015; Gao et al., 2016; Gharahkhani et al., 2019; Hagg et al., 2015; Hartwig et al., 2016; Howe et al., 2017; Huang et al., 2016; Jacobs et al., 2020; Lyall et al., 2017; Shu et al., 2019; Speed et al., 2019; Taylor et al., 2019; Taylor et al., 2016; Todd et al., 2015; Wade, Chiesa, et al., 2018; Wang, Cheng, et al., 2018; Zeng et al., 2019) | 13 | (Censin et al., 2017; Chen, Fan, Huang, et al., 2019; Dale et al., 2017; Dusingize et al., 2020; Gianfrancesco, Glymour, et al., 2017; Guo et al., 2017; Holmes et al., 2014; Jarvis et al., 2016; Liu et al., 2020; Ooi et al., 2019; Sun et al., 2019; Wade, Carslake, et al., 2018; Wang, Lu, et al., 2018) |
| GRS/multiple IVs vs. single SNP(s) | 5 | 5 | (Budu-Aggrey et al., 2019; Colak et al., 2016; Eriksson et al., 2017; Fall et al., 2015; Richmond et al., 2014) | 0 |  |
| Two subsets of SNPs grouping SNPs with the same biological pathway on the exposure | 1 | 0 |  | 1 | (Martins-Silva et al., 2019) |
| Detection of outlier/influential SNP(s): Cook’s distance, Studentized residuals, HEIDI-outlier*,* and leave-one-out analyses | 15 | 4 | (Corbin et al., 2016; Takahashi et al., 2019; Wurtz et al., 2014; Zhou et al., 2019) | 13 | (Bae and Lee, 2019; Corbin et al., 2016; Hartwig et al., 2016; Jacobs et al., 2020; Liu et al., 2020; Martins-Silva et al., 2019; Noyce et al., 2017; Ooi et al., 2019; Takahashi et al., 2019; van den Broek et al., 2018; Zeng et al., 2019; Zhang et al., 2020; Zhao, Xu, et al., 2020) |
| **Methods to detect associations between IVs and the outcome outside of the pathway through the exposure** | **85** | **30** |  | **61** |  |
| Estimating the associations between the IVs and measured risk factors for the outcome | 75 | 19 | (Benn et al., 2016; Budu-Aggrey et al., 2019; Carreras-Torres et al., 2016; Carreras-Torres et al., 2018; Davies et al., 2015; Gharahkhani et al., 2019; Howe et al., 2017; Kaltoft et al., 2020; Kemp et al., 2016; Kurz and Laxy, 2020; Lyall et al., 2017; Lyngdoh et al., 2012; Shu et al., 2019; Todd et al., 2015; Wade, Carslake, et al., 2018; Wang, Cheng, et al., 2018; Winter-Jensen et al., 2020; Zeng et al., 2019; Zhou et al., 2019) | 56 | (Afzal et al., 2014; Barning and Abarin, 2016; Brennan et al., 2009; Censin et al., 2017; Censin et al., 2019; Chen, Fan, Huang, et al., 2019; Chen, Fan, Yang, et al., 2019; Colak et al., 2016; Dale et al., 2017; Dixon et al., 2016; Eriksson et al., 2017; Fan et al., 2018; Geng et al., 2018; Gianfrancesco, Glymour, et al., 2017; Granell et al., 2014; Guo et al., 2017; Guo et al., 2016; Hagg et al., 2015; Holmes et al., 2014; Howe et al., 2019; Huang et al., 2016; Hung et al., 2014; Jokela et al., 2012; Kivimäki et al., 2011; Kivimaki et al., 2008; Klovaite et al., 2015; Lawlor et al., 2011; Lee et al., 2018; Lee et al., 2019; Lewis et al., 2010; Mao et al., 2017; Nordestgaard et al., 2012; Nordestgaard et al., 2017; Qian, Wang, et al., 2019; Reed et al., 2017; Richmond et al., 2014; Rode et al., 2014; Schnurr et al., 2018; Shungin et al., 2015; Skaaby et al., 2018; Stender et al., 2013; Sun et al., 2020; Sun et al., 2019; Tan et al., 2019; Thakkinstian et al., 2014; Thrift et al., 2015; Thrift et al., 2014; Timpson et al., 2009; Timpson et al., 2011; Tyrrell et al., 2019; Varbo et al., 2015; Wade, Chiesa, et al., 2018; Walter, Kubzansky, et al., 2015; Wang, Lu, et al., 2018; Warodomwichit et al., 2013; Wurtz et al., 2014) |
| Documenting the associations between the IV and risk factors for the outcome in the literature | 7 | 5 | (Carreras-Torres et al., 2016; Fall et al., 2013; Gao et al., 2016; Kaltoft et al., 2020; Wade, Carslake, et al., 2018) | 2 | (Chatterjee et al., 2017; Jarvis et al., 2016) |
| Adjusting IV-outcome or IV-confounders associations for exposure | 18 | 7 | (Chatterjee et al., 2017; Kivimäki et al., 2011; Painter et al., 2016; Robinson et al., 2020; Timpson et al., 2009; Wang, Cheng, et al., 2018; Wurtz et al., 2014) | 11 | (Cole et al., 2016; Eriksson et al., 2017; Gianfrancesco, Glymour, et al., 2017; Huang et al., 2016; Jokela et al., 2012; Kivimaki et al., 2008; Lawlor et al., 2011; Panoutsopoulou et al., 2014; Tyrrell et al., 2019; Walter, Glymour, et al., 2015; Walter, Kubzansky, et al., 2015) |
| Adjusting IV-outcome association or MR analyses for covariates potentially involved in pleiotropic pathways^5^ | 5 | 2 | (Chatterjee et al., 2017; Kaltoft et al., 2020) | 3 | (Censin et al., 2019; Guo et al., 2016; Sun et al., 2020) |
| Comparison of the exposure-outcome conventional vs. IV estimated effects | 4 | 1 | (Painter et al., 2016) | 3 | (Kivimäki et al., 2011; Walter, Glymour, et al., 2015; Walter, Kubzansky, et al., 2015) |
| Comparison of the IV-outcome vs. IV-exposure associations | 2 | 2 | (Barning and Abarin, 2016; Tyrrell et al., 2019) | 0 |  |
| Estimating the association between the IV and the outcome | 2 | 1 | (Speed et al., 2019) | 1 | (Wang, Lu, et al., 2018) |
| Mediation analysis estimating the direct effect of the IV on the outcome | 3 | 0 |  | 3 | (Afzal et al., 2014; Fan et al., 2018; Gianfrancesco, Glymour, et al., 2017) |
| **Other methods** | **5** | **0** |  | **5** |  |
| The use of positive or negative control outcomes | 3 | 0 |  | 3 | (Nordestgaard et al., 2017; Richardson et al., 2020; Tyrrell et al., 2019) |
| Colocalization | 1 | 0 |  | 1 | (Liu et al., 2020) |
| Verifying the concordance of MR results with those from other studies (MR, clinical trial) | 1 | 0 |  | 1 | (Holmes et al., 2014) |
| **No method reported for assessment of independence or exclusion restriction assumptions** | 8 | (den Hollander et al., 2017; Gianfrancesco, Stridh, et al., 2017; Lim et al., 2009; Mumby et al., 2011; Reed et al., 2020; Vimaleswaran et al., 2013; Wang et al., 2016; Welsh et al., 2010) | | | |
| **Abbreviations:** IV, instrumental variable; MR, mendelian randomization; PRESSO, pleiotropy residual sum and outlier; SNP, single*-*nucleotide polymorphism; RAPS, Robust Adjusted Prole Score; IVW, inverse-variance weighted; *FTO*, fat mass and obesity-associated; GRS, genetic risk score; HEIDI, Heterogeneity in Dependent Instruments.  ^1^Many studies cited more than one method and thus the number of methods reported in the Table exceed the total number of studies (n=128).  ^2^See (Burgess et al., 2020) and (Burgess et al., 2015) for a summary of the listed methods.  ^3^Of the 69 studies that reported using MR-Egger, 66 used the intercept test p-value to infer whether or not pleiotropy was present, two studies (Fan et al., 2018; Tyrrell et al., 2016) compared the MR-Egger slope and the conventional MR causal effect estimate, while the last study (Chen, Fan, Yang, et al., 2019) did not specify how the MR-Egger results were used.  ^4^One study (Censin et al., 2017) did not specify the heterogeneity test used.  ^5^Three articles (Censin et al., 2019; Guo et al., 2016; Sun et al., 2020) mentioned adjustment of MR analyses for covariates potentially involved in pleiotropic pathways without specifying as multivariable MR. | | | | | |

**Table S6. Summary description of methods cited for detection or accounting the violation of the independence and/or restriction exclusion assumptions in the review.**

Note: Entries from this table are drawn from several publications (Burgess et al., 2017; Burgess et al., 2020; Burgess and Thompson, 2017; Hemani et al., 2018; Slob and Burgess, 2020). References are provided for a more detailed descriptions of each method.

| **Methods** | **Description** | **Main assumptions and/or limitations^1^** | **References** |
| --- | --- | --- | --- |
| **Robust and other IV estimation methods** | | | |
| Robust methods |  |  |  |
| MR-Egger | Regression of SNPs-outcome on SNPs-exposure effects weighted by the inverse-variance of the SNPs-outcome associations, allowing a non-zero intercept. The intercept of the regression may be interpreted as the estimated average pleiotropic effect, and the slope as the pleiotropy-adjusted causal effect estimates. | Assumes the InSIDE assumption (independence between the SNPs-exposure and the direct SNPs-outcome effects)^2^, estimates are sensitive to outliers and influential datapoints, low precision of the intercept and causal estimates compared to methods like IVW, increased type 1 error if InSIDE is not satisfied. | (Bowden et al., 2015; Burgess et al., 2017; Burgess et al., 2020) |
| Median-based methods | Estimates the causal effect as the median of the of the SNPs-specific causal effect estimates. | Assumes the majority valid assumption (at least 50% of the SNPs are valid), likely to be inefficient when the individual SNP estimates receive equal weight in the analysis, may be affected by addition or removal of SNPs, increased type 1 error if there exist an asymmetric distribution of the SNP pleiotropic effects around zero. | (Bowden, Davey Smith, et al., 2016; Burgess et al., 2017; Burgess et al., 2020) |
| Mode-based methods | Estimates the causal effect as the mode of the smoothed empirical density function of the SNPs-specific causal effect estimates. | Assumes the plurality valid assumption (more SNPs estimate the true causal effect than estimate any other quantity), sensitive to bandwidth parameter required for density estimation, may be affected by addition or removal of SNPs, low power. | (Burgess et al., 2020; Carreras-Torres et al., 2018; Hartwig et al., 2017) |
| MR-PRESSO | Performs the IVW method using all SNPs and omitting each SNP in turn, and then excludes the SNP(s) whose omission showed a significant decrease in the residual sum of squares (used as a measure of heterogeneity of SNP ratio estimates). | Higher false positive rate if there are many invalid SNPs. | (Slob and Burgess, 2020; Verbanck et al., 2018) |
| MR-RAPS | Robust Adjusted Profile Score: Models the distribution of the pleiotropic effects of SNPs using a random-effect distribution. | Assumes the pleiotropic effects of SNPs to be symmetric around zero, may be affected by violations of balanced pleiotropy assumption. | (Slob and Burgess, 2020; Zhao, Wang, et al., 2020) |
| Other IV estimation methods |  |  |  |
| Multivariable MR | Extension of MR analysis that estimates the direct causal effect of several risk factors on a single outcome using multiple SNPs. | Requires that each SNP be associated with at least one exposure and not affect the outcome other than through these exposures, does not address unmeasured pleiotropy, weak instrument bias may be substantial if large numbers of SNPs used. | (Burgess et al., 2020; Burgess and Thompson, 2015; Sanderson et al., 2019) |
| Inverse-variance weighted | Regression of SNPs-outcome on SNPs-exposure associations, weighted by the inverse of variance of SNPs-outcome associations, allowing a null intercept. | Assumes all SNPs to be valid, biased if average pleiotropic effect differs from zero, vulnerable to weak instrument bias, more efficient than MR-Egger. | (Bowden et al., 2015; Bowden, Del Greco M, et al., 2016; Burgess et al., 2013) |
| Likelihood-based methods | SNP effects on exposure and outcome are modelled as a bivariate normal distribution and estimated using maximum likelihood estimation or Bayesian methods. | Assumes linear relationship between the exposure and the outcome, and a bivariate normal distribution for the SNPs-exposure and SNPs-outcome associations, computationally complex. | (Burgess et al., 2013; Burgess et al., 2016; Burgess et al., 2015) |
| **Methods to detect heterogeneity of estimated causal effects across IVs** | | | |
| Graphical assessment: scatter plots, forest plots, funnel plots, leave-one-out plots, and histograms | Visual examination of whether the causal effects estimates from different IVs are similar. Scatter plots show the SNPs-outcome vs. SNPs-exposure effects, with the linear estimation of the overall causal effect. Forest plots show the causal effect of each SNP as well as the overall causal estimate. Leave-one-out plots show the overall polygenic causal estimate as well as the re-estimated causal effect after sequential removing of one SNP at a time. Funnel plots show the SNP strength vs. the causal estimate from each SNP. Histograms show the distribution of estimates from the leave-one out analysis. | Subjective interpretation of heterogeneity. | (Burgess et al., 2017; Burgess et al., 2020; Carreras-Torres et al., 2018; Hemani et al., 2018; Zhou et al., 2019) |
| Statistical criteria and tests: I^2^, r^2^, H statistic, Cochran's Q, Rucker's Q, over-identification tests | Statistical tests of heterogeneity of IV-specific causal effects. | Low power for detecting heterogeneity when the number of IVs is small, overestimation of heterogeneity in presence of all valid IVs with different magnitudes of causal effect. | (Bowden et al., 2017; Burgess et al., 2017; Glymour et al., 2012; Hartwig et al., 2016; Hemani et al., 2018; Zhou et al., 2019) |
| Comparisons of estimated MR causal effects across IVs (GRS or multiple IVs) |  |  |  |
| Before and after exclusion of SNPs suspected of pleiotropy | Evaluate whether the causal effects estimated from polygenic MR analyses before and after exclusion of SNP(s) suspected of pleiotropy are consistent. | Subjective interpretation of consistency. | (Carreras-Torres et al., 2016; Hemani et al., 2018) |
| GRS or multiple IVs vs. single SNP(s) | Evaluate whether the causal effect estimated from a polygenic MR analysis is consistent with that using single SNP(s). | Subjective interpretation of consistency. | (Colak et al., 2016; Fall et al., 2015; Hemani et al., 2018) |
| Two subsets of SNPs grouping SNPs with the same biological pathway on the exposure | Evaluate whether the causal effects estimated from MR analyses using two subsets of SNPs, each grouping SNPs with the same biological pathway on the exposure, are consistent. | Subjective interpretation of consistency. | (Hemani et al., 2018; Martins-Silva et al., 2019) |
| Detection of outlier/influential SNP(s): Cook’s distance, Studentized residuals, HEIDI-outlier, leave-one-out analyses | Detect outlier or influential SNP(s). Cook’s distance is a measure of leverage, allowing to detect SNPs that exerted a disproportionately large influence on the overall causal effect. A Studentized residual is the ratio between a residual from the regression model and an estimate of its standard error, indicating the goodness-of-fit in the model for that point. The leave-one-out analysis evaluates whether the overall polygenic causal effect estimate is similar with the re-estimated causal effect after sequential removing of one SNP at a time. HEIDI-outlier uses linkage disequilibrium information to test whether there exist heterogeneity in the causal effects estimated from near-independent SNPs and exclude from analysis SNP(s) that have apparent pleiotropic effects on both exposure and outcome. | Cut-off values to determine influential SNPs are sensitive to sample size and may require subjective interpretation, HEIDI-outlier may not detect certainly pleiotropic outlier SNPs (e.g. those with very small effects). | (Burgess and Thompson, 2017; Hemani et al., 2018; Verbanck et al., 2018; Zhou et al., 2019; Zhu et al., 2016) |
| **Methods to detect associations between IVs and the outcome outside of the pathway through the exposure** | | | |
| Estimating the associations between the IVs and measured risk factors for the outcome | Statistically significant associations between IVs and measured covariates may be indicative of violation of MR assumptions. | Does not account for unmeasured covariates, significant association with a covariate may be due to other causes than pleiotropy (e.g., mediation, population stratification, selection/collider bias), non-significant association may be due to low power, statistical significance threshold may be difficult to set in case of large number if IVs. | **(Burgess et al., 2017; Burgess et al., 2020; Wade, Carslake, et al., 2018)** |
| Documenting the associations between the IV and risk factors for the outcome in the literature | Documents reported associations between the IVs and covariates that may affect the outcome of interest which may be indicative of violation of MR assumptions. | Does not account for unmeasured covariates, significant association with a covariate may be due to other causes than pleiotropy (e.g. mediation, population stratification, selection/collider bias). | (Fall et al., 2013; Wade, Carslake, et al., 2018) |
| Adjusting IV-outcome or IV-confounders associations for exposure | Consists of assessing whether the association IV-outcome or IV-confounders is attenuated by adjustment for the exposure as would be expected if the effect of the IV on the outcome or on a confounder is mediated through the exposure. | Subjective interpretation of effect attenuation, does not account for unmeasured confounders. | (Walter, Glymour, et al., 2015) |
| Adjusting IV-outcome association or MR analyses for covariates potentially involved in pleiotropic pathways | Consists in assessing whether the association IV-outcome is attenuated by adjustment for a covariate associated with the outcome, as would be expected if the effect of the IV on the outcome is mediated through this covariate. | Subjective interpretation of effect attenuation, does not account for unmeasured confounders, may be difficult to distinguish vertical to horizontal pleiotropy. | (Chatterjee et al., 2017) |
|  | Account pleiotropy by adjusting MR analyses for covariates potentially involved in pleiotropic pathways. | Requires individual-level data, does not deal with unmeasured confounders. | **(Burgess et al., 2017)** |
| Comparison of the exposure-outcome conventional vs. IV estimated effects | The IV is suspected to be invalid if the effect of the exposure on the outcome estimated by ordinary least squares is greater than that estimated by MR. | Assumes positive exposure-outcome unmeasured confounding, performs better in case of pleiotropy by direct effect of the IV on the outcome than by indirect effect via confounders, may detects valid IVs as invalids (false-positive rate). | (Burgess, 2012; Glymour et al., 2012) |
| Comparison of the IV-outcome vs. IV-exposure associations | Consists in justifying the non-violation of exclusion restriction given that the IVs are associated with exposure and not with the outcome. | A null association between the IV and the outcome may exist despite violation of exclusion restriction (e.g. when the direct and indirect effects of the IV on the outcome are opposite and symmetrical to zero, the total effect estimated by regressing the outcome on the IV may be non-significant). | (Barning and Abarin, 2016) |
|  | Consists in suspecting the presence of pleiotropy given that the SNPs have larger effects on the outcome than their effects on the exposure. | SNP-exposure and SNP-outcome effects may be difficult to compare if evaluate on different scales. | (Tyrrell et al., 2019) |
| Estimating the association between the IV and the outcome | Suspects the violation of the exclusion restriction assumption by detecting significant associations between the SNPs and the outcome of interest. | A significant association between the SNPs and the outcome may be due to vertical pleiotropy. | (Speed et al., 2019; Wang, Lu, et al., 2018) |
| Mediation analysis estimating the direct effect of the IV on the outcome | Decomposes the total effect of the IV on the outcome into an indirect effect via the exposure and a direct effect (independent of exposure or pleiotropic). | Assumes no IV-outcome, exposure-outcome, and IV-exposure confounding; no exposure-outcome confounders affected by the IV, may be biased if unmanaged IV-exposure interaction, does not apply to unmeasured confounders. | (VanderWeele, 2016) |
| **Other methods** | | | |
| The use of positive or negative control outcomes | Assesses whether there is a genetic association between the exposure and an outcome for which one is certain that the exposure is causal (positive control), or for which one is not thought to be affected by the exposure (negative control). | Adequate negative and positive controls may be difficult to find, a significant genetic association may not be indicative of pleiotropy. | (Burgess and Davey Smith, 2017; Burgess et al., 2020) |
| Colocalization | Aims to detect whether a shared causal SNP between the two traits (exposure and outcome) is plausible. | **Low statistical power, may be inexact if there exist multiple conditionally independent causal variants in the cis region, or if the exposure and outcome trait effects were estimated in populations with different linkage desequilibrium patterns.** | **(Burgess et al., 2020; Giambartolomei et al., 2014; Hemani et al., 2018; Liu et al., 2020)** |
| Verifying the concordance of MR results with those from other studies (MR, clinical trial) | Justify the whether the exclusion restriction assumption is not violated based on the concordance between its results and those of previous MR studies using different genetic variants and of a clinical trial. | **Concordance may be due to chance, different populations , different methods, depends on the quality of the study compared to.** | (Holmes et al., 2014) |
| Abbreviations: MR, mendelian randomization; IV, instrumental variable; SNP, single-nucleotide polymorphism; InSIDE, Instrument Strength Independent of Direct Effect; IVW, inverse-variance weighted; HEIDI, heterogeneity in dependent instruments; PRESSO, Pleiotropy Residual Sum and Outlier; RAPS, Robust Adjusted Profile Score.  ^1^All the methods are vulnerable to measurement errors.  ^2^The MR-egger intercept test does not rely to the InSIDE assumption, but the estimation of the slope as well as the interpretation of the intercept coefficient as the average pleiotropic effect require that InSIDE be satisfied (Burgess and Thompson, 2017). | | | |

**Table S7. Discussion of the independence and/or exclusion restriction assumptions.**

| **Discussion of the independence and/or exclusion restriction assumptions** | **n** | **References** |
| --- | --- | --- |
| **Plausibility of the independence and/or exclusion restriction assumptions** | **n=128** |  |
| Discussed with specific reference to pleiotropy | 89 | (Bae and Lee, 2019; Benn et al., 2016; Brower et al., 2019; Budu-Aggrey et al., 2019; Carreras-Torres et al., 2016; Censin et al., 2017; Chatterjee et al., 2017; Chen, Fan, Huang, et al., 2019; Chen, Fan, Yang, et al., 2019; Colak et al., 2016; Cole et al., 2016; Corbin et al., 2016; Dale et al., 2017; Davies et al., 2015; Dixon et al., 2016; Dusingize et al., 2020; Eriksson et al., 2017; Fall et al., 2013; Fall et al., 2015; Gao et al., 2016; Geng et al., 2018; Granell et al., 2014; Guo et al., 2016; Hagg et al., 2015; Hartwig et al., 2016; Holmes et al., 2014; Howe et al., 2019; Huang et al., 2016; Jacobs et al., 2020; Jarvis et al., 2016; Jokela et al., 2012; Kaltoft et al., 2020; Kemp et al., 2016; Kivimäki et al., 2011; Larsson et al., 2020; Larsson et al., 2018; Lee et al., 2018; Lee et al., 2019; Lewis et al., 2010; Lindstrom et al., 2017; Liu et al., 2020; Lyall et al., 2017; Lyngdoh et al., 2012; Mao et al., 2017; Marini et al., 2020; Martins-Silva et al., 2019; Mokry et al., 2016; Mukherjee et al., 2015; Mumby et al., 2011; Nordestgaard et al., 2012; Noyce et al., 2017; Ooi et al., 2019; Painter et al., 2016; Reed et al., 2017; Richardson et al., 2020; Richmond et al., 2014; Robinson et al., 2020; Schnurr et al., 2018; Shu et al., 2019; Shungin et al., 2015; Skaaby et al., 2018; Speed et al., 2019; Stender et al., 2013; Sun et al., 2020; Sun et al., 2019; Takahashi et al., 2019; Tan et al., 2019; Taylor et al., 2019; Taylor et al., 2016; Thrift et al., 2015; Thrift et al., 2014; Timpson et al., 2009; Timpson et al., 2011; Todd et al., 2015; Tyrrell et al., 2016; Varbo et al., 2015; Vimaleswaran et al., 2013; Wade, Carslake, et al., 2018; Wade, Chiesa, et al., 2018; Wang, Cheng, et al., 2018; Welsh et al., 2010; Winter-Jensen et al., 2020; Wurtz et al., 2014; Xu et al., 2017; Xu et al., 2019; Zeng et al., 2019; Zhang et al., 2020; Zhao, Xu, et al., 2020; Zhou et al., 2019) |
| Discussed without specific reference to pleiotropy | 19 | (Afzal et al., 2014; Busch et al., 2019; Carreras-Torres et al., 2018; Censin et al., 2019; Gianfrancesco, Glymour, et al., 2017; Hung et al., 2014; Kivimaki et al., 2008; Klovaite et al., 2015; Kurz and Laxy, 2020; Lawlor et al., 2011; Panoutsopoulou et al., 2014; Qian, Wang, et al., 2019; Rode et al., 2014; Thakkinstian et al., 2014; Tyrrell et al., 2019; Walter, Glymour, et al., 2015; Walter, Kubzansky, et al., 2015; Wang, Lu, et al., 2018; Warodomwichit et al., 2013) |
| **IV invalidity^1^** | **n=128** |  |
| Suspected with specific reference to pleiotropy | 16 | (Censin et al., 2017; Corbin et al., 2016; Gharahkhani et al., 2019; Howe et al., 2019; Kivimäki et al., 2011; Larsson et al., 2020; Painter et al., 2016; Shu et al., 2019; Speed et al., 2019; Sun et al., 2020; Takahashi et al., 2019; van den Broek et al., 2018; Wade, Carslake, et al., 2018; Wang, Cheng, et al., 2018; Winter-Jensen et al., 2020; Xu et al., 2019) |
| Suspected without specific reference to pleiotropy | 5 | (Afzal et al., 2014; Gianfrancesco, Glymour, et al., 2017; Walter, Glymour, et al., 2015; Walter, Kubzansky, et al., 2015; Wang, Lu, et al., 2018) |
| **Impact of IV invalidity on the validity of MR results** | **n=21** |  |
| May have affected validity of results | 8 | (Censin et al., 2017; Corbin et al., 2016; Howe et al., 2019; Kivimäki et al., 2011; Painter et al., 2016; van den Broek et al., 2018; Walter, Glymour, et al., 2015; Walter, Kubzansky, et al., 2015) |
| No or low impact on validity of results | 9 | (Gharahkhani et al., 2019; Gianfrancesco, Glymour, et al., 2017; Shu et al., 2019; Speed et al., 2019; Takahashi et al., 2019; Wade, Carslake, et al., 2018; Wang, Lu, et al., 2018; Winter-Jensen et al., 2020; Xu et al., 2019) |
| Impact not (clearly) reported | 4 | (Afzal et al., 2014; Larsson et al., 2020; Sun et al., 2020; Wang, Cheng, et al., 2018) |
| **Suspicion of invalidity/pleiotropy of *FTO* SNP(s)** | **n=128** |  |
| Yes | 6 | (Gianfrancesco, Glymour, et al., 2017; Kivimäki et al., 2011; van den Broek et al., 2018; Walter, Glymour, et al., 2015; Walter, Kubzansky, et al., 2015; Wang, Cheng, et al., 2018) |
| ***FTO* SNP(s) suspected to be invalid/pleiotropic** | **n=6** |  |
| rs1558902^2^ | 3 | (Gianfrancesco, Glymour, et al., 2017; Walter, Glymour, et al., 2015; Walter, Kubzansky, et al., 2015) |
| rs1421085^3^ | 2 | (Kivimäki et al., 2011; van den Broek et al., 2018) |
| rs17817449^4^ | 1 | (Wang, Cheng, et al., 2018) |
| **Abbreviations:** IV, instrumental variable; MR, mendelian randomization; SNP, single*-*nucleotide polymorphism; *FTO*, fat mass and obesity-associated.  ^1^Refers to the suspicion of invalidity of one or more body mass index IV(s) for any outcome of interest.  ^2^The outcomes of interest involved in the suspected invalidity of rs1558902 were multiple sclerosis susceptibility (Gianfrancesco, Glymour, et al., 2017), phobic anxiety symptoms (Walter, Glymour, et al., 2015), and depression (Walter, Kubzansky, et al., 2015).  ^3^The outcomes of interest involved in the suspected pleiotropy of rs1421085 were common mental disorders (Kivimäki et al., 2011), and subjective well-being (van den Broek et al., 2018).  ^4^The outcome of interest involved in the suspected pleiotropy of rs17817449 was lipid profiles (Wang, Cheng, et al., 2018). | | |

**Table S8. List of 21 studies that suspected IV pleiotropy/invalidity^1^**

| **Studies** | **SNP suspected of pleiotropy /invalidity** | **Chr^2^** | **Nearest gene^3^** | **Variable suspected in a pathway from the IV to the outcome outside of the exposure pathway, if any^4^** | **Outcome(s)^5^** |
| --- | --- | --- | --- | --- | --- |
| Afzal et al., 2014 | Not reported | - | - | NA | Type 2 diabetes |
| Censin et al., 2017 | rs13130484 | 4 | *GNPDA2* | Education | Type 1 diabetes |
|  | rs13107325 | 4 | *SLC39A8* | Education |  |
| Corbin et al., 2016 | rs7903146 | 10 | *TCF7L2* | NA | Type 2 diabetes |
| Gharahkhani et al., 2019 | Not reported | - | - | Smoking, alcohol intake, coffee/tea consumption, height | Overall cancer risk and mortality, and specific type cancer risk |
| Gianfrancesco, Glymour, et al., 2017 | rs11126666 | 2 | *KCNK3* | NA | Multiple sclerosis |
|  | rs2112347 | 5 | *POC5* |  |  |
|  | rs1558902 | 16 | *FTO* |  |  |
|  | rs7243357 | 18 | *GRP* |  |  |
|  | rs7599312 | 2 | *ERBB4* |  |  |
| Howe et al., 2019 | Not reported | - | - | NA | Education |
| Kivimäki et al., 2011 | rs1421085 | 16 | *FTO* | NA | Common mental disorders |
| Larsson et al., 2020 | Not reported | - | - | NA | Coronary artery disease, atrial fibrillation, and transient ischaemic attack |
| Painter et al., 2016 | rs2075650 | 19 | *TOMM40* | NA | Endometrial cancer |
| Shu et al., 2019 | rs1800437 | 19 | *GIPR* | 2-h glucose | Breast cancer risk |
|  | rs1801282 | 3 | *PPARG* | Fasting insulin |  |
|  | rs2176040 | 2 | *NYAP2, MIR5702* | Fasting insulin |  |
|  | rs7903146 | 10 | *TCF7L2* | Fasting glucose, fasting insulin |  |
| Speed et al., 2019 | Not reported | - | - | NA | Depression |
| Sun et al., 2020 | Not reported | - | - | NA | Doctor-diagnosed active asthma |
| Takahashi et al., 2019 | rs1814170 | 7 | *ZC3HAV1* | NA | Meningioma |
|  | rs3803286 | 14 | *TRAF3* |  |  |
|  | rs7084454 | 10 | *MLLT10* |  |  |
| van den Broek et al., 2018 | rs1421085 | 16 | *FTO* | NA | Subjective well-being |
|  | rs943005 | 6 | *FTH1P5, RPS17P5* |  |  |
|  | rs13021737 | 2 | *TMEM18, LINC01875* |  |  |
| Wade, Carslake, et al., 2018 | rs17024393 | 1 | *GNAT2* | NA | Mortality from other cancers |
|  | rs13107325 | 4 | *SLC39A8* |  |  |
|  | rs17001654 | 4 | *SCARB2* |  |  |
|  | rs1167827 | 7 | *HIP1* |  |  |
|  | rs11030104 | 11 | *BDNF* |  |  |
|  | rs3888190 | 16 | *ATP2A1* |  |  |
|  | rs2075650 | 19 | *TOMM40* |  |  |
| Walter, Glymour, et al., 2015 | rs1558902 | 16 | *FTO* | NA | Phobic anxiety symptoms |
| Walter, Kubzansky, et al., 2015 | rs1558902 | 16 | *FTO* | NA | Depression |
| Wang, Cheng, et al., 2018 | rs4776970 | 15 | *MAP2K5* | Blood pressure | Lipid profiles |
|  | rs12597579 | 16 | *SNRPEP3, GP2* | Blood pressure |  |
|  | rs17817449 | 16 | *FTO* | Blood pressure, age |  |
|  | rs261967 | 5 | *PCSK1, CAST* | Blood pressure |  |
|  | rs4715210 | 6 | *FTH1P5* | Blood pressure, fasting plasma glucose, age |  |
|  | rs6567160 | 18 | *MC4R* | Blood pressure |  |
|  | rs574367 | 1 | *SEC16B* | Blood pressure |  |
|  | rs6545814 | 2 | *ADCY3* | Fasting plasma glucose |  |
| Wang, Lu, et al., 2018**^6^** | Not reported | - | - | Lipid profile, hypertension | Blood lead level |
| Winter-Jensen et al., 2020 | Not reported | - | - | Smoking status | Hospital contacts related to infections |
| Xu et al., 2019 | rs2287019 | 19 | *QPCTL* | NA | Asthma |
| Abbreviations: IV, instrumental variable; SNPs, single nucleotide polymorphisms; Chr, chromosome; NA, not applicable.  ^1^ Corresponds to studies that suspected invalidity of the IV using MR assumption assessment methods, regardless of whether there was an impact on the validity of the results.  ^2^ When not explicitly reported in the manuscript, the chromosome was identified by searching the SNP in GWAS catalog (<https://www.ebi.ac.uk/gwas/>).  ^3^ When not explicitly reported in the manuscript, the nearest gene was identified by searching in GWAS catalog (<https://www.ebi.ac.uk/gwas/>). In instances where a SNP appeared close to two genes, we reported both.  ^4^ Applicable to studies that reported associations between the IVs and measured covariates.  ^5^ If several outcomes of interest were considered in the study, we reported only that (those) suspected of being affected by the SNP irrespective of the pathway via exposure.  ^6^ When discussing the validity of the exclusion restriction assumption, the study mentioned 3 SNPs (rs11671664, rs261967, and rs3932549) associated with the outcome of interest, but whose exclusion did not change the results. | | | | | |

# **References**

Afzal, S., Brøndum-Jacobsen, P., Bojesen, S. E., and Nordestgaard, B. G. (2014). Vitamin D concentration, obesity, and risk of diabetes: a mendelian randomisation study. *Lancet Diabetes Endocrinol.* 2, 298-306. doi:10.1016/S2213-8587(13)70200-6

Bae, S.-C., and Lee, Y. H. (2019). Causal association between body mass index and risk of rheumatoid arthritis: A Mendelian randomization study. *Eur. J. Clin. Invest.* 49, e13076. doi:10.1111/eci.13076

Barning, F., and Abarin, T. (2016). Assessing the Causality Factors in the Association between (Abdominal) Obesity and Physical Activity among the Newfoundland Population-A Mendelian Randomization Analysis. *Genet. Epigenet.* 8, 15-24. doi:10.4137/GEG.S38289

Benn, M., Tybjærg-Hansen, A., Davey Smith, G., and Nordestgaard, B. G. (2016). High body mass index and cancer risk-a Mendelian randomisation study. *Eur. J. Epidemiol.* 31, 879-892. doi:10.1007/s10654-016-0147-5

Boef, A. G. C., Dekkers, O. M., and le Cessie, S. (2015). Mendelian randomization studies: a review of the approaches used and the quality of reporting. *Int. J. Epidemiol.* 44, 496-511. doi:10.1093/ije/dyv071

Bonnefond, A., Yengo, L., Dechaume, A., Canouil, M., Castelain, M., Roger, E., et al. (2017). Relationship between salivary/pancreatic amylase and body mass index: a systems biology approach. *BMC Med.* 15, 37. doi:10.1186/s12916-017-0784-x

Bowden, J., Davey Smith, G., and Burgess, S. (2015). Mendelian randomization with invalid instruments: effect estimation and bias detection through Egger regression. *Int. J. Epidemiol.* 44, 512-525. doi:10.1093/ije/dyv080

Bowden, J., Davey Smith, G., Haycock, P. C., and Burgess, S. (2016). Consistent Estimation in Mendelian Randomization with Some Invalid Instruments Using a Weighted Median Estimator. *Genet. Epidemiol.* 40, 304-314. doi:10.1002/gepi.21965

Bowden, J., Del Greco M, F., Minelli, C., Davey Smith, G., Sheehan, N., and Thompson, J. (2017). A framework for the investigation of pleiotropy in two‐sample summary data Mendelian randomization. *Stat. Med.* 36, 1783-1802. doi:10.1002/sim.7221

Bowden, J., Del Greco M, F., Minelli, C., Davey Smith, G., Sheehan, N. A., and Thompson, J. R. (2016). Assessing the suitability of summary data for two-sample Mendelian randomization analyses using MR-Egger regression: the role of the I 2 statistic. *Int. J. Epidemiol.* 45, 1961-1974. doi:10.1093/ije/dyw220

Brennan, P., McKay, J., Moore, L., Zaridze, D., Mukeria, A., Szeszenia-Dabrowska, N., et al. (2009). Obesity and cancer: Mendelian randomization approach utilizing the FTO genotype. *Int. J. Epidemiol.* 38, 971-975. doi:10.1093/ije/dyp162

Brower, M. A., Hai, Y., Jones, M. R., Guo, X., Chen, Y. D. I., Rotter, J. I., et al. (2019). Bidirectional Mendelian randomization to explore the causal relationships between body mass index and polycystic ovary syndrome. *Hum. Reprod.* 34, 127-136. doi:10.1093/humrep/dey343

Budu-Aggrey, A., Brumpton, B., Tyrrell, J., Watkins, S., Modalsli, E. H., Celis-Morales, C., et al. (2019). Evidence of a causal relationship between body mass index and psoriasis: A mendelian randomization study. *PLoS Med.* 16, e1002739. doi:10.1371/journal.pmed.1002739

Burgess, S. (2012). Re: "credible mendelian randomization studies: Approaches for evaluating the instrumental variable assumptions". *Am. J. Epidemiol.* 176, 456-457. doi:10.1093/aje/kws249

Burgess, S., Bowden, J., Fall, T., Ingelsson, E., and Thompson, S. G. (2017). Sensitivity analyses for robust causal inference from Mendelian randomization analyses with multiple genetic variants. *Epidemiology* 28, 30-42. doi:10.1097/EDE.0000000000000559

Burgess, S., Butterworth, A., and Thompson, S. G. (2013). Mendelian randomization analysis with multiple genetic variants using summarized data. *Genet. Epidemiol.* 37, 658-665. doi:10.1002/gepi.21758

Burgess, S., and Davey Smith, G. (2017). Mendelian randomization implicates high-density lipoprotein cholesterol–associated mechanisms in etiology of age-related macular degeneration. *Ophthalmology* 124, 1165-1174. doi:10.1016/j.ophtha.2017.03.042

Burgess, S., Davey Smith, G., Davies, N. M., Dudbridge, F., Gill, D., Glymour, M. M., et al. (2020). Guidelines for performing Mendelian randomization investigations. *Wellcome Open Res.* 4, 186. doi:10.12688/wellcomeopenres.15555.2

Burgess, S., Dudbridge, F., and Thompson, S. G. (2016). Combining information on multiple instrumental variables in Mendelian randomization: comparison of allele score and summarized data methods. *Stat. Med.* 35, 1880-1906. doi:10.1002/sim.6835

Burgess, S., Scott, R. A., Timpson, N. J., Davey Smith, G., Thompson, S. G., and Epic- InterAct Consortium (2015). Using published data in Mendelian randomization: a blueprint for efficient identification of causal risk factors. *Eur. J. Epidemiol.* 30, 543-552. doi:10.1007/s10654-015-0011-z

Burgess, S., and Thompson, S. G. (2015). Multivariable Mendelian randomization: the use of pleiotropic genetic variants to estimate causal effects. *Am. J. Epidemiol.* 181, 251-260. doi:10.1093/aje/kwu283

Burgess, S., and Thompson, S. G. (2017). Interpreting findings from Mendelian randomization using the MR-Egger method. *Eur. J. Epidemiol.* 32, 377-389. doi:10.1007/s10654-017-0255-x

Busch, A. S., Hollis, B., Day, F. R., Sorensen, K., Aksglaede, L., Perry, J. R. B., et al. (2019). Voice break in boys-temporal relations with other pubertal milestones and likely causal effects of BMI. *Hum. Reprod.* 34, 1514-1522. doi:10.1093/humrep/dez118

Carreras-Torres, R., Haycock, P. C., Relton, C. L., Martin, R. M., Smith, G. D., Kraft, P., et al. (2016). The causal relevance of body mass index in different histological types of lung cancer: A Mendelian randomization study. *Sci. Rep.* 6, 31121. doi:10.1038/srep31121

Carreras-Torres, R., Johansson, M., Haycock, P. C., Relton, C. L., Smith, G. D., Brennan, P., et al. (2018). Role of obesity in smoking behaviour: Mendelian randomisation study in UK Biobank. *Br. Med. J.* 361, k1767. doi:10.1136/bmj.k1767

Censin, J. C., Nowak, C., Cooper, N., Bergsten, P., Todd, J. A., and Fall, T. (2017). Childhood adiposity and risk of type 1 diabetes: A Mendelian randomization study. *PLoS Med.* 14, e1002362. doi:10.1371/journal.pmed.1002362

Censin, J. C., Peters, S. A. E., Bovijn, J., Ferreira, T., Pulit, S. L., Magi, R., et al. (2019). Causal relationships between obesity and the leading causes of death in women and men. *PLoS Genet.* 15, e1008405. doi:10.1371/journal.pgen.1008405

Chatterjee, N. A., Giulianini, F., Geelhoed, B., Lunetta, K. L., Misialek, J. R., Niemeijer, M. N., et al. (2017). Genetic Obesity and the Risk of Atrial Fibrillation Causal Estimates from Mendelian Randomization. *Circulation* 135, 741-754. doi:10.1161/circulationaha.116.024921

Chen, Y.-C., Fan, H.-Y., Huang, Y.-T., Huang, S.-Y., Liou, T.-H., and Lee, Y. L. (2019). Causal relationships between adiposity and childhood asthma: bi-directional Mendelian Randomization analysis. *Int. J. Obes. (Lond.)* 43, 73-81. doi:10.1038/s41366-018-0160-8

Chen, Y.-C., Fan, H.-Y., Yang, C., Hsieh, R.-H., Pan, W.-H., and Lee, Y. L. (2019). Assessing causality between childhood adiposity and early puberty: A bidirectional Mendelian randomization and longitudinal study. *Metabolism* 100, 153961. doi:10.1016/j.metabol.2019.153961

Colak, Y., Afzal, S., Lange, P., and Nordestgaard, B. G. (2016). Obese individuals experience wheezing without asthma but not asthma without wheezing: a Mendelian randomisation study of 85 437 adults from the Copenhagen General Population Study. *Thorax* 71, 247-254. doi:10.1136/thoraxjnl-2015-207379

Cole, C. B., Nikpay, M., Stewart, A. F., and McPherson, R. (2016). Increased genetic risk for obesity in premature coronary artery disease. *Eur. J. Hum. Genet.* 24, 587-591. doi:10.1038/ejhg.2015.162

Corbin, L. J., Richmond, R. C., Wade, K. H., Burgess, S., Bowden, J., Smith, G. D., et al. (2016). BMI as a Modifiable Risk Factor for Type 2 Diabetes: Refining and Understanding Causal Estimates Using Mendelian Randomization. *Diabetes* 65, 3002-3007. doi:10.2337/db16-0418

Dale, C. E., Fatemifar, G., Palmer, T. M., White, J., Prieto-Merino, D., Zabaneh, D., et al. (2017). Causal Associations of Adiposity and Body Fat Distribution With Coronary Heart Disease, Stroke Subtypes, and Type 2 Diabetes Mellitus: A Mendelian Randomization Analysis. *Circulation* 135, 2373-2388. doi:10.1161/CIRCULATIONAHA.116.026560

Davies, N. M., Gaunt, T. R., Lewis, S. J., Holly, J., Donovan, J. L., Hamdy, F. C., et al. (2015). The effects of height and BMI on prostate cancer incidence and mortality: a Mendelian randomization study in 20,848 cases and 20,214 controls from the PRACTICAL consortium. *Cancer Causes Control* 26, 1603-1616. doi:10.1007/s10552-015-0654-9

den Hollander, W. J., Broer, L., Schurmann, C., Meyre, D., den Hoed, C. M., Mayerle, J., et al. (2017). Helicobacter pylori colonization and obesity - a Mendelian randomization study. *Sci. Rep.* 7, 14467. doi:10.1038/s41598-017-14106-4

Dixon, S. C., Nagle, C. M., Thrift, A. P., Pharoah, P. D., Pearce, C. L., Zheng, W., et al. (2016). Adult body mass index and risk of ovarian cancer by subtype: a Mendelian randomization study. *Int. J. Epidemiol.* 45, 884-895. doi:10.1093/ije/dyw158

Dusingize, J. C., Olsen, C. M., An, J., Pandeya, N., Law, M. H., Thompson, B. S., et al. (2020). Body mass index and height and risk of cutaneous melanoma: Mendelian randomization analyses. *Int. J. Epidemiol.* 49, 1236-1245. doi:10.1093/ije/dyaa009

Eriksson, J., Haring, R., Grarup, N., Vandenput, L., Wallaschofski, H., Lorentzen, E., et al. (2017). Causal relationship between obesity and serum testosterone status in men: A bidirectional mendelian randomization analysis. *PLoS One* 12, e0176277. doi:10.1371/journal.pone.0176277

Fall, T., Hagg, S., Magi, R., Ploner, A., Fischer, K., Horikoshi, M., et al. (2013). The Role of Adiposity in Cardiometabolic Traits: A Mendelian Randomization Analysis. *PLoS Med.* 10, e1001474. doi:10.1371/journal.pmed.1001474

Fall, T., Hagg, S., Ploner, A., Magi, R., Fischer, K., Draisma, H. H., et al. (2015). Age- and sex-specific causal effects of adiposity on cardiovascular risk factors. *Diabetes* 64, 1841-1852. doi:10.2337/db14-0988

Fan, H. Y., Huang, Y. T., Hsieh, R. H., Chao, J. C. J., Tung, Y. C., Lee, Y. L., et al. (2018). Birthweight, time-varying adiposity growth and early menarche in girls: A Mendelian randomisation and mediation analysis. *Obes. Res. Clin. Pract.* 12, 445-451. doi:10.1016/j.orcp.2018.07.008

Fussey, J. M., Beaumont, R. N., Wood, A. R., Vaidya, B., Smith, J., and Tyrrell, J. (2020). Does obesity cause thyroid cancer? A Mendelian randomization study. *J. Clin. Endocrinol. Metab.* 105, e2398-e2407. doi:10.1210/clinem/dgaa250

Gao, C., Patel, C. J., Michailidou, K., Peters, U., Gong, J., Schildkraut, J., et al. (2016). Mendelian randomization study of adiposity-related traits and risk of breast, ovarian, prostate, lung and colorectal cancer. *Int. J. Epidemiol.* 45, 896-908. doi:10.1093/ije/dyw129

Geng, T., Smith, C. E., Li, C., and Huang, T. (2018). Childhood BMI and adult type 2 diabetes, coronary artery diseases, chronic kidney disease, and cardiometabolic traits: A Mendelian randomization analysis. *Diabetes Care* 41, 1089-1096. doi:10.2337/dc17-2141/-/DC1

Gharahkhani, P., Ong, J. S., An, J. Y., Law, M. H., Whiteman, D. C., Neale, R. E., et al. (2019). Effect of increased body mass index on risk of diagnosis or death from cancer. *Br. J. Cancer* 120, 565-570. doi:10.1038/s41416-019-0386-9

Giambartolomei, C., Vukcevic, D., Schadt, E. E., Franke, L., Hingorani, A. D., Wallace, C., et al. (2014). Bayesian test for colocalisation between pairs of genetic association studies using summary statistics. *PLoS Genet.* 10, e1004383. doi:10.1371/journal.pgen.1004383

Gianfrancesco, M. A., Glymour, M. M., Walter, S., Rhead, B., Shao, X., Shen, L., et al. (2017). Causal Effect of Genetic Variants Associated With Body Mass Index on Multiple Sclerosis Susceptibility. *Am. J. Epidemiol.* 185, 162-171. doi:10.1093/aje/kww120

Gianfrancesco, M. A., Stridh, P., Rhead, B., Shao, X. R., Xu, E., Graves, J. S., et al. (2017). Evidence for a causal relationship between low vitamin D, high BMI, and pediatric-onset MS. *Neurology* 88, 1623-1629. doi:10.1212/WNL.0000000000003849

Glymour, M. M., Tchetgen Tchetgen, E. J., and Robins, J. M. (2012). Credible mendelian randomization studies: Approaches for evaluating the instrumental variable assumptions. *Am. J. Epidemiol.* 175, 332-339. doi:10.1093/aje/kwr323

Granell, R., Henderson, A. J., Evans, D. M., Smith, G. D., Ness, A. R., Lewis, S., et al. (2014). Effects of BMI, fat mass, and lean mass on asthma in childhood: a Mendelian randomization study. *PLoS Med.* 11, e1001669. doi:10.1371/journal.pmed.1001669

Guo, Q., Burgess, S., Turman, C., Bolla, M. K., Wang, Q., Lush, M., et al. (2017). Body mass index and breast cancer survival: a Mendelian randomization analysis. *Int. J. Epidemiol.* 46, 1814-1822. doi:10.1093/ije/dyx131

Guo, Y., Andersen, S. W., Shu, X. O., Michailidou, K., Bolla, M. K., Wang, Q., et al. (2016). Genetically Predicted Body Mass Index and Breast Cancer Risk: Mendelian Randomization Analyses of Data from 145,000 Women of European Descent. *PLoS Med.* 13, e1002105. doi:10.1371/journal.pmed.1002105

Hagg, S., Fall, T., Ploner, A., Magi, R., Fischer, K., Draisma, H. H. M., et al. (2015). Adiposity as a cause of cardiovascular disease: A Mendelian randomization study. *Int. J. Epidemiol.* 44, 578-586. doi:10.1093/ije/dyv094

Hartwig, F. P., Bowden, J., de Mola, C. L., Tovo-Rodrigues, L., Smith, G. D., and Horta, B. L. (2016). Body mass index and psychiatric disorders: a Mendelian randomization study. *Sci. Rep.* 6, 32730. doi:10.1038/srep32730

Hartwig, F. P., Davey Smith, G., and Bowden, J. (2017). Robust inference in summary data Mendelian randomization via the zero modal pleiotropy assumption. *Int. J. Epidemiol.* 46, 1985-1998. doi:10.1093/ije/dyx102

Hemani, G., Bowden, J., and Davey Smith, G. (2018). Evaluating the potential role of pleiotropy in Mendelian randomization studies. *Hum. Mol. Genet.* 27, R195-R208. doi:10.1093/hmg/ddy163

Holmes, M. V., Lange, L. A., Palmer, T., Lanktree, M. B., North, K. E., Almoguera, B., et al. (2014). Causal effects of body mass index on cardiometabolic traits and events: a Mendelian randomization analysis. *Am. J. Hum. Genet.* 94, 198-208. doi:10.1016/j.ajhg.2013.12.014

Howe, L. D., Kanayalal, R., Harrison, S., Beaumont, R. N., Davies, A. R., Frayling, T. M., et al. (2019). Effects of body mass index on relationship status, social contact and socio-economic position: Mendelian randomization and within-sibling study in UK Biobank. *Int. J. Epidemiol.* 49, 1173-1184. doi:10.1093/ije/dyz240

Howe, L. J., Trela-Larsen, L., Taylor, M., Heron, J., Munafo, M. R., and Taylor, A. E. (2017). Body mass index, body dissatisfaction and adolescent smoking initiation. *Drug Alcohol Depend.* 178, 143-149. doi:10.1016/j.drugalcdep.2017.04.008

Huang, Y., Xu, M., Xie, L., Wang, T., Huang, X., Lv, X., et al. (2016). Obesity and peripheral arterial disease: A Mendelian Randomization analysis. *Atherosclerosis* 247, 218-224. doi:10.1016/j.atherosclerosis.2015.12.034

Hung, C. F., Rivera, M., Craddock, N., Owen, M. J., Gill, M., Korszun, A., et al. (2014). Relationship between obesity and the risk of clinically significant depression: Mendelian randomisation study. *Br. J. Psychiatry* 205, 24-28. doi:10.1192/bjp.bp.113.130419

Jacobs, B. M., Noyce, A. J., Giovannoni, G., and Dobson, R. (2020). BMI and low vitamin D are causal factors for multiple sclerosis: A Mendelian Randomization study. *Neurol. Neuroimmunol. Neuroinflamm.* 7, e662. doi:10.1212/NXI.0000000000000662

Jarvis, D., Mitchell, J. S., Law, P. J., Palin, K., Tuupanen, S., Gylfe, A., et al. (2016). Mendelian randomisation analysis strongly implicates adiposity with risk of developing colorectal cancer. *Br. J. Cancer* 115, 266-272. doi:10.1038/bjc.2016.188

Jokela, M., Elovainio, M., Keltikangas-Järvinen, L., Batty, G. D., Hintsanen, M., Seppälä, I., et al. (2012). Body mass index and depressive symptoms: instrumental-variables regression with genetic risk score. *Genes Brain Behav.* 11, 942-948. doi:10.1111/j.1601-183X.2012.00846.x

Kaltoft, M., Langsted, A., and Nordestgaard, B. G. (2020). Obesity as a Causal Risk Factor for Aortic Valve Stenosis. *J. Am. Coll. Cardiol.* 75, 163-176. doi:10.1016/j.jacc.2019.10.050

Kemp, J. P., Sayers, A., Davey Smith, G., Tobias, J. H., and Evans, D. M. (2016). Using Mendelian randomization to investigate a possible causal relationship between adiposity and increased bone mineral density at different skeletal sites in children. *Int. J. Epidemiol.* 45, 1560-1572. doi:10.1093/ije/dyw079

Kivimäki, M., Jokela, M., Hamer, M., Geddes, J., Ebmeier, K., Kumari, M., et al. (2011). Examining overweight and obesity as risk factors for common mental disorders using fat mass and obesity-associated (FTO) genotype-instrumented analysis: The Whitehall II Study, 1985-2004. *Am. J. Epidemiol.* 173, 421-429. doi:10.1093/aje/kwq444

Kivimaki, M., Smith, G. D., Timpson, N. J., Lawlor, D. A., Batty, G. D., Kahonen, M., et al. (2008). Lifetime body mass index and later atherosclerosis risk in young adults: examining causal links using Mendelian randomization in the Cardiovascular Risk in Young Finns study. *Eur. Heart J.* 29, 2552-2560. doi:10.1093/eurheartj/ehn252

Klovaite, J., Benn, M., and Nordestgaard, B. G. (2015). Obesity as a causal risk factor for deep venous thrombosis: a Mendelian randomization study. *J. Intern. Med.* 277, 573-584. doi:10.1111/joim.12299

Kurz, C. F., and Laxy, M. (2020). Application of Mendelian Randomization to Investigate the Association of Body Mass Index with Health Care Costs. *Med. Decis. Making* 40, 156-169. doi:10.1177/0272989X20905809

Larsson, S. C., Back, M., Rees, J. M. B., Mason, A. M., and Burgess, S. (2020). Body mass index and body composition in relation to 14 cardiovascular conditions in UK Biobank: a Mendelian randomization study. *Eur. Heart J.* 41, 221-226. doi:10.1093/eurheartj/ehz388

Larsson, S. C., Burgess, S., and Michaëlsson, K. (2018). Genetic association between adiposity and gout: a Mendelian randomization study. *Rheumatology* 57, 2145-2148. doi:10.1093/rheumatology/key229

Lawlor, D. A., Harbord, R. M., Tybjaerg-Hansen, A., Palmer, T. M., Zacho, J., Benn, M., et al. (2011). Using genetic loci to understand the relationship between adiposity and psychological distress: A Mendelian Randomization study in the Copenhagen General Population Study of 53221 adults. *J. Intern. Med.* 269, 525-537. doi:10.1111/j.1365-2796.2011.02343.x

Lee, M. R., Lim, Y. H., and Hong, Y. C. (2018). Causal association of body mass index with hypertension using a Mendelian randomization design. *Medicine (Baltimore)* 97, e11252. doi:10.1097/MD.0000000000011252

Lee, S. J., Lee, J. Y., and Sung, J. (2019). Obesity and Bone Health Revisited: A Mendelian Randomization Study for Koreans. *J. Bone Miner. Res.* 34, 1058-1067. doi:10.1002/jbmr.3678

Lewis, S. J., Murad, A., Chen, L. N., Davey Smith, G., Donovan, J., Palmer, T., et al. (2010). Associations between an Obesity Related Genetic Variant (FTO rs9939609) and Prostate Cancer Risk. *PLoS One* 5, e13485. doi:10.1371/journal.pone.0013485

Lim, L. S., Tai, E. S., Aung, T., Tay, W. T., Saw, S. M., Seielstad, M., et al. (2009). Relation of age-related cataract with obesity and obesity genes in an Asian population. *Am. J. Epidemiol.* 169, 1267-1274. doi:10.1093/aje/kwp045

Lindstrom, S., Germain, M., Crous-Bou, M., Smith, E. N., Morange, P. E., Vlieg, A. V., et al. (2017). Assessing the causal relationship between obesity and venous thromboembolism through a Mendelian Randomization study. *Hum. Genet.* 136, 897-902. doi:10.1007/s00439-017-1811-x

Liu, Q., Pan, J., Berzuini, C., Rutter, M. K., and Guo, H. (2020). Integrative analysis of Mendelian randomization and Bayesian colocalization highlights four genes with putative BMI-mediated causal pathways to diabetes. *Sci. Rep.* 10, 7476. doi:10.1038/s41598-020-64493-4

Lyall, D. M., Celis-Morales, C., Ward, J., Iliodromiti, S., Anderson, J. J., Gill, J. M. R., et al. (2017). Association of Body Mass Index With Cardiometabolic Disease in the UK Biobank A Mendelian Randomization Study. *JAMA Cardiol.* 2, 882-889. doi:10.1001/jamacardio.2016.5804

Lyngdoh, T., Vuistiner, P., Marques-Vidal, P., Rousson, V., Waeber, G., Vollenweider, P., et al. (2012). Serum uric acid and adiposity: deciphering causality using a bidirectional Mendelian randomization approach. *PLoS One* 7, e39321. doi:10.1371/journal.pone.0039321

Mao, Y., Yan, C., Lu, Q., Zhu, M., Yu, F., Wang, C., et al. (2017). Genetically predicted high body mass index is associated with increased gastric cancer risk. *Eur. J. Hum. Genet.* 25, 1061-1066. doi:10.1038/ejhg.2017.103

Marini, S., Merino, J., Montgomery, B. E., Malik, R., Sudlow, C. L., Dichgans, M., et al. (2020). Mendelian Randomization Study of Obesity and Cerebrovascular Disease. *Ann. Neurol.* 87, 516-524. doi:10.1002/ana.25686

Martins-Silva, T., Vaz, J. S., Hutz, M. H., Salatino-Oliveira, A., Genro, J. P., Hartwig, F. P., et al. (2019). Assessing causality in the association between attention-deficit/hyperactivity disorder and obesity: a Mendelian randomization study. *Int. J. Obes. (Lond.)* 43, 2500-2508. doi:10.1038/s41366-019-0346-8

Mokry, L. E., Ross, S., Timpson, N. J., Sawcer, S., Smith, G. D., and Richards, J. B. (2016). Obesity and Multiple Sclerosis: A Mendelian Randomization Study. *PLoS Med.* 13, e1002053. doi:10.1371/journal.pmed.1002053

Mukherjee, S., Walter, S., Kauwe, J. S. K., Saykin, A. J., Bennett, D. A., Larson, E. B., et al. (2015). Genetically predicted body mass index and Alzheimer's disease-related phenotypes in three large samples: Mendelian randomization analyses. *Alzheimers Dement.* 11, 1439-1451. doi:10.1016/j.jalz.2015.05.015

Mumby, H. S., Elks, C. E., Li, S., Sharp, S. J., Khaw, K. T., Luben, R. N., et al. (2011). Mendelian Randomisation Study of Childhood BMI and Early Menarche. *J. Obes.* 2011, 180729. doi:10.1155/2011/180729

Nordestgaard, B. G., Palmer, T. M., Benn, M., Zacho, J., Tybaerg-Hansen, A., Smith, G. D., et al. (2012). The Effect of Elevated Body Mass Index on Ischemic Heart Disease Risk: Causal Estimates from a Mendelian Randomisation Approach. *PLoS Med.* 9, e1001212. doi:10.1371/journal.pmed.1001212

Nordestgaard, L. T., Tybjaerg-Hansen, A., Nordestgaard, B. G., and Frikke-Schmidt, R. (2017). Body Mass Index and Risk of Alzheimer's Disease: A Mendelian Randomization Study of 399,536 Individuals. *J. Clin. Endocrinol. Metab.* 102, 2310-2320. doi:10.1210/jc.2017-00195

Noyce, A. J., Kia, D. A., Hemani, G., Nicolas, A., Price, T. R., De Pablo-Fernandez, E., et al. (2017). Estimating the causal influence of body mass index on risk of Parkinson disease: A Mendelian randomisation study. *PLoS Med.* 14, e1002314. doi:10.1371/journal.pmed.1002314

Ooi, B. N. S., Loh, H., Ho, P. J., Milne, R. L., Giles, G., Gao, C., et al. (2019). The genetic interplay between body mass index, breast size and breast cancer risk: a Mendelian randomization analysis. *Int. J. Epidemiol.* 48, 781-794. doi:10.1093/ije/dyz124

Painter, J. N., O'Mara, T. A., Marquart, L., Webb, P. M., Attia, J., Medland, S. E., et al. (2016). Genetic Risk Score Mendelian Randomization Shows that Obesity Measured as Body Mass Index, but not Waist:Hip Ratio, Is Causal for Endometrial Cancer. *Cancer Epidemiol. Biomarkers Prev.* 25, 1503-1510. doi:10.1158/1055-9965.EPI-16-0147

Panoutsopoulou, K., Metrustry, S., Doherty, S. A., Laslett, L. L., Maciewicz, R. A., Hart, D. J., et al. (2014). The effect of FTO variation on increased osteoarthritis risk is mediated through body mass index: a Mendelian randomisation study. *Ann. Rheum. Dis.* 73, 2082-2086. doi:10.1136/annrheumdis-2013-203772

Qian, F., Rookus, M. A., Leslie, G., Risch, H. A., Greene, M. H., Aalfs, C. M., et al. (2019). Mendelian randomisation study of height and body mass index as modifiers of ovarian cancer risk in 22,588 BRCA1 and BRCA2 mutation carriers. *Br. J. Cancer* 121, 180-192. doi:10.1038/s41416-019-0492-8

Qian, F., Wang, S. F., Mitchell, J., McGuffog, L., Barrowdale, D., Leslie, G., et al. (2019). Height and Body Mass Index as Modifiers of Breast Cancer Risk in BRCA1/2 Mutation Carriers: A Mendelian Randomization Study. *J. Natl. Cancer Inst.* 111, 350-364. doi:10.1093/jnci/djy132

Reed, Z. E., Micali, N., Bulik, C. M., Davey Smith, G., and Wade, K. H. (2017). Assessing the causal role of adiposity on disordered eating in childhood, adolescence, and adulthood: a Mendelian randomization analysis. *Am. J. Clin. Nutr.* 106, 764-772. doi:10.3945/ajcn.117.154104

Reed, Z. E., Suderman, M. J., Relton, C. L., Davis, O. S. P., and Hemani, G. (2020). The association of DNA methylation with body mass index: distinguishing between predictors and biomarkers. *Clin. Epigenetics* 12, 50. doi:10.1186/s13148-020-00841-5

Richardson, T. G., Sanderson, E., Elsworth, B., Tilling, K., and Davey Smith, G. (2020). Use of genetic variation to separate the effects of early and later life adiposity on disease risk: mendelian randomisation study. *Br. Med. J.* 369, m1203. doi:10.1136/bmj.m1203

Richmond, R. C., Davey Smith, G., Ness, A. R., den Hoed, M., McMahon, G., and Timpson, N. J. (2014). Assessing Causality in the Association between Child Adiposity and Physical Activity Levels: A Mendelian Randomization Analysis. *PLoS Med.* 11, e1001618. doi:10.1371/journal.pmed.1001618

Robinson, J. R., Carroll, R. J., Bastarache, L., Chen, Q., Mou, Z., Wei, W. Q., et al. (2020). Association of Genetic Risk of Obesity with Postoperative Complications Using Mendelian Randomization. *World J. Surg.* 44, 84-94. doi:10.1007/s00268-019-05202-9

Rode, L., Nordestgaard, B. G., Weischer, M., and Bojesen, S. E. (2014). Increased Body Mass Index, Elevated C-reactive Protein, and Short Telomere Length. *J. Clin. Endocrinol. Metab.* 99, E1671-E1675. doi:10.1210/jc.2014-1161

Sanderson, E., Davey Smith, G., Windmeijer, F., and Bowden, J. (2019). An examination of multivariable Mendelian randomization in the single-sample and two-sample summary data settings. *Int. J. Epidemiol.* 48, 713-727. doi:10.1093/ije/dyy262

Schnurr, T. M., Viitasalo, A., Eloranta, A. M., Damsgaard, C. T., Mahendran, Y., Have, C. T., et al. (2018). Genetic predisposition to adiposity is associated with increased objectively assessed sedentary time in young children. *Int. J. Obes. (Lond.)* 42, 111-114. doi:10.1038/ijo.2017.235

Shu, X., Wu, L., Khankari, N. K., Shu, X. O., Wang, T. J., Michailidou, K., et al. (2019). Associations of obesity and circulating insulin and glucose with breast cancer risk: a Mendelian randomization analysis. *Int. J. Epidemiol.* 48, 795-806. doi:10.1093/ije/dyy201

Shungin, D., Cornelis, M. C., Divaris, K., Holtfreter, B., Shaffer, J. R., Yu, Y. H., et al. (2015). Using genetics to test the causal relationship of total adiposity and periodontitis: Mendelian randomization analyses in the Gene-Lifestyle Interactions and Dental Endpoints (GLIDE) Consortium. *Int. J. Epidemiol.* 44, 638-650. doi:10.1093/ije/dyv075

Skaaby, T., Taylor, A. E., Thuesen, B. H., Jacobsen, R. K., Friedrich, N., Møllehave, L. T., et al. (2018). Estimating the causal effect of body mass index on hay fever, asthma and lung function using Mendelian randomization. *Allergy* 73, 153-164. doi:10.1111/all.13242

Slob, E. A., and Burgess, S. (2020). A comparison of robust Mendelian randomization methods using summary data. *Genet. Epidemiol.* 44, 313-329. doi:10.1002/gepi.22295

Speed, M. S., Jefsen, O. H., Borglum, A. D., Speed, D., and Ostergaard, S. D. (2019). Investigating the association between body fat and depression via Mendelian randomization. *Transl. Psychiatry* 9, 184. doi:10.1038/s41398-019-0516-4

Stender, S., Nordestgaard, B. G., and Tybjaerg-Hansen, A. (2013). Elevated body mass index as a causal risk factor for symptomatic gallstone disease: a Mendelian randomization study. *Hepatology* 58, 2133-2141. doi:10.1002/hep.26563

Sun, Y. Q., Brumpton, B., Langhammer, A., Chen, Y., Kvaloy, K., and Mai, X. M. (2020). Adiposity and asthma in adults: a bidirectional Mendelian randomisation analysis of The HUNT Study. *Thorax* 75, 202-208. doi:10.1136/thoraxjnl-2019-213678

Sun, Y. Q., Burgess, S., Staley, J. R., Wood, A. M., Bell, S., Kaptoge, S. K., et al. (2019). Body mass index and all cause mortality in HUNT and UK Biobank studies: linear and non-linear mendelian randomisation analyses. *Br. Med. J.* 364, l1042. doi:10.1136/bmj.l1042

Takahashi, H., Cornish, A. J., Sud, A., Law, P. J., Disney-Hogg, L., Calvocoressi, L., et al. (2019). Mendelian randomization provides support for obesity as a risk factor for meningioma. *Sci. Rep.* 9, 309. doi:10.1038/s41598-018-36186-6

Tan, A. G., Kifley, A., Flood, V. M., Holliday, E. G., Scott, R. J., Cumming, R. G., et al. (2019). Evaluating the associations between obesity and age-related cataract: a Mendelian randomization study. *Am. J. Clin. Nutr.* 110, 969-976. doi:10.1093/ajcn/nqz167

Taylor, A. E., Richmond, R. C., Palviainen, T., Loukola, A., Wootton, R. E., Kaprio, J., et al. (2019). The effect of body mass index on smoking behaviour and nicotine metabolism: a Mendelian randomization study. *Hum. Mol. Genet.* 28, 1322-1330. doi:10.1093/hmg/ddy434

Taylor, P. N., Richmond, R., Davies, N., Sayers, A., Stevenson, K., Woltersdorf, W., et al. (2016). Paradoxical Relationship Between Body Mass Index and Thyroid Hormone Levels: A Study Using Mendelian Randomization. *J. Clin. Endocrinol. Metab.* 101, 730-738. doi:10.1210/jc.2015-3505

Thakkinstian, A., Chailurkit, L., Warodomwichit, D., Ratanachaiwong, W., Yamwong, S., Chanprasertyothin, S., et al. (2014). Causal relationship between body mass index and fetuin-A level in the asian population: a bidirectional mendelian randomization study. *Clin. Endocrinol. (Oxf.)* 81, 197-203. doi:10.1111/cen.12303

Thrift, A. P., Gong, J., Peters, U., Chang-Claude, J., Rudolph, A., Slattery, M. L., et al. (2015). Mendelian Randomization Study of Body Mass Index and Colorectal Cancer Risk. *Cancer Epidemiol. Biomarkers Prev.* 24, 1024-1031. doi:10.1158/1055-9965.EPI-14-1309

Thrift, A. P., Shaheen, N. J., Gammon, M. D., Bernstein, L., Reid, B. J., Onstad, L., et al. (2014). Obesity and Risk of Esophageal Adenocarcinoma and Barrett's Esophagus: A Mendelian Randomization Study. *J. Natl. Cancer Inst.* 106, dju252. doi:10.1093/jnci/dju252

Timpson, N. J., Harbord, R., Davey Smith, G., Zacho, J., Tybjaerg-Hansen, A., and Nordestgaard, B. G. (2009). Does greater adiposity increase blood pressure and hypertension risk?: Mendelian randomization using the FTO/MC4R genotype. *Hypertension* 54, 84-90. doi:10.1161/HYPERTENSIONAHA.109.130005

Timpson, N. J., Nordestgaard, B. G., Harbord, R. M., Zacho, J., Frayling, T. M., Tybjærg-Hansen, A., et al. (2011). C-reactive protein levels and body mass index: elucidating direction of causation through reciprocal Mendelian randomization. *Int. J. Obes. (Lond.)* 35, 300-308. doi:10.1038/ijo.2010.137

Todd, J. N., Dahlström, E. H., Salem, R. M., Sandholm, N., Forsblom, C., McKnight, A. J., et al. (2015). Genetic Evidence for a Causal Role of Obesity in Diabetic Kidney Disease. *Diabetes* 64, 4238-4246. doi:10.2337/db15-0254

Tyrrell, J., Jones, S. E., Beaumont, R., Astley, C. M., Lovell, R., Yaghootkar, H., et al. (2016). Height, body mass index, and socioeconomic status: mendelian randomisation study in UK Biobank. *Br. Med. J.* 352, i582. doi:10.1136/bmj.i582

Tyrrell, J., Mulugeta, A., Wood, A. R., Zhou, A., Beaumont, R. N., Tuke, M. A., et al. (2019). Using genetics to understand the causal influence of higher BMI on depression. *Int. J. Epidemiol.* 48, 834-848. doi:10.1093/ije/dyy223

van 't Hof, F. N. G., Vaucher, J., Holmes, M. V., de Wilde, A., Baas, A. F., Blankensteijn, J. D., et al. (2017). Genetic variants associated with type 2 diabetes and adiposity and risk of intracranial and abdominal aortic aneurysms. *Eur. J. Hum. Genet.* 25, 758-762. doi:10.1038/ejhg.2017.48

van den Broek, N., Treur, J. L., Larsen, J. K., Verhagen, M., Verweij, K. J. H., and Vink, J. M. (2018). Causal associations between body mass index and mental health: a Mendelian randomisation study. *J. Epidemiol. Community Health* 72, 708-710. doi:10.1136/jech-2017-210000

VanderWeele, T. J. (2016). Mediation analysis: a practitioner's guide. *Annu. Rev. Public Health* 37, 17-32. doi:10.1146/annurev-publhealth-032315-021402

Varbo, A., Benn, M., Smith, G. D., Timpson, N. J., Tybjaerg-Hansen, A., and Nordestgaard, B. G. (2015). Remnant cholesterol, low-density lipoprotein cholesterol, and blood pressure as mediators from obesity to ischemic heart disease. *Circ. Res.* 116, 665-673. doi:10.1161/CIRCRESAHA.116.304846

Vasan, S. K., Noordam, R., Gowri, M. S., Neville, M. J., Karpe, F., and Christodoulides, C. (2019). The proposed systemic thermogenic metabolites succinate and 12,13-diHOME are inversely associated with adiposity and related metabolic traits: evidence from a large human cross-sectional study. *Diabetologia* 62, 2079-2087. doi:10.1007/s00125-019-4947-5

Verbanck, M., Chen, C.-Y., Neale, B., and Do, R. (2018). Detection of widespread horizontal pleiotropy in causal relationships inferred from Mendelian randomization between complex traits and diseases. *Nat. Genet.* 50, 693-698. doi:10.1038/s41588-018-0099-7

Vimaleswaran, K. S., Berry, D. J., Lu, C., Tikkanen, E., Pilz, S., Hiraki, L. T., et al. (2013). Causal relationship between obesity and vitamin D status: bi-directional Mendelian randomization analysis of multiple cohorts. *PLoS Med.* 10, e1001383. doi:10.1371/journal.pmed.1001383

Wade, K. H., Carslake, D., Sattar, N., Davey Smith, G., and Timpson, N. J. (2018). BMI and Mortality in UK Biobank: Revised Estimates Using Mendelian Randomization. *Obesity (Silver Spring, Md.)* 26, 1796-1806. doi:10.1002/oby.22313

Wade, K. H., Chiesa, S. T., Hughes, A. D., Chaturvedi, N., Charakida, M., Rapala, A., et al. (2018). Assessing the causal role of body mass index on cardiovascular health in young adults: Mendelian randomization and recall-by-genotype analyses. *Circulation* 138, 2187-2201. doi:10.1161/CIRCULATIONAHA.117.033278

Wainberg, M., Mahajan, A., Kundaje, A., McCarthy, M. I., Ingelsson, E., Sinnott-Armstrong, N., et al. (2019). Homogeneity in the association of body mass index with type 2 diabetes across the UK Biobank: A Mendelian randomization study. *PLoS Med.* 16, e1002982. doi:10.1371/journal.pmed.1002982

Walter, S., Glymour, M. M., Koenen, K., Liang, L., Tchetgen Tchetgen, E. J., Cornelis, M., et al. (2015). Do genetic risk scores for body mass index predict risk of phobic anxiety? Evidence for a shared genetic risk factor. *Psychol. Med.* 45, 181-191. doi:10.1017/S0033291714001226

Walter, S., Kubzansky, L. D., Koenen, K. C., Liang, L., Tchetgen Tchetgen, E. J., Cornelis, M. C., et al. (2015). Revisiting Mendelian randomization studies of the effect of body mass index on depression. *Am. J. Med. Genet. B Neuropsychiatr. Genet.* 168B, 108-115. doi:10.1002/ajmg.b.32286

Wang, N., Cheng, J., Ning, Z., Chen, Y., Han, B., Li, Q., et al. (2018). Type 2 Diabetes and Adiposity Induce Different Lipid Profile Disorders: A Mendelian Randomization Analysis. *J. Clin. Endocrinol. Metab.* 103, 2016-2025. doi:10.1210/jc.2017-02789

Wang, N., Lu, M., Chen, C., Xia, F., Han, B., Li, Q., et al. (2018). Adiposity genetic risk score modifies the association between blood lead level and body mass index. *J. Clin. Endocrinol. Metab.* 103, 4005-4013. doi:10.1210/jc.2018-00472

Wang, T., Ma, X., Tang, T., Jin, L., Peng, D., Zhang, R., et al. (2016). Overall and central obesity with insulin sensitivity and secretion in a Han Chinese population: a Mendelian randomization analysis. *Int. J. Obes. (Lond.)* 40, 1736-1741. doi:10.1038/ijo.2016.155

Wang, T., Zhang, R., Ma, X. J., Wang, S. Y., He, Z., Huang, Y. P., et al. (2018). Causal Association of Overall Obesity and Abdominal Obesity with Type 2 Diabetes: A Mendelian Randomization Analysis. *Obesity (Silver Spring, Md.)* 26, 934-942. doi:10.1002/oby.22167

Warodomwichit, D., Sritara, C., Thakkinstian, A., Chailurkit, L. O., Yamwong, S., Ratanachaiwong, W., et al. (2013). Causal inference of the effect of adiposity on bone mineral density in adults. *Clin. Endocrinol. (Oxf.)* 78, 694-699. doi:10.1111/cen.12061

Welsh, P., Polisecki, E., Robertson, M., Jahn, S., Buckley, B. M., de Craen, A. J., et al. (2010). Unraveling the directional link between adiposity and inflammation: a bidirectional Mendelian randomization approach. *J. Clin. Endocrinol. Metab.* 95, 93-99. doi:10.1210/jc.2009-1064

Winter-Jensen, M., Afzal, S., Jess, T., Nordestgaard, B. G., and Allin, K. H. (2020). Body mass index and risk of infections: a Mendelian randomization study of 101,447 individuals. *Eur. J. Epidemiol.* 35, 347-354. doi:10.1007/s10654-020-00630-7

Wurtz, P., Wang, Q., Kangas, A. J., Richmond, R. C., Skarp, J., Tiainen, M., et al. (2014). Metabolic signatures of adiposity in young adults: Mendelian randomization analysis and effects of weight change. *PLoS Med.* 11, e1001765. doi:10.1371/journal.pmed.1001765

Xu, L., Borges, M. C., Hemani, G., and Lawlor, D. A. (2017). The role of glycaemic and lipid risk factors in mediating the effect of BMI on coronary heart disease: a two-step, two-sample Mendelian randomisation study. *Diabetologia* 60, 2210-2220. doi:10.1007/s00125-017-4396-y

Xu, S., Gilliland, F. D., and Conti, D. V. (2019). Elucidation of causal direction between asthma and obesity: a bi-directional Mendelian randomization study. *Int. J. Epidemiol.* 48, 899-907. doi:10.1093/ije/dyz070

Zeng, P., Yu, X., and Xu, H. (2019). Association Between Premorbid Body Mass Index and Amyotrophic Lateral Sclerosis: Causal Inference Through Genetic Approaches. *Front. Neurol.* 10, 543. doi:10.3389/fneur.2019.00543

Zhang, L., Tang, L., Huang, T., and Fan, D. (2020). Life Course Adiposity and Amyotrophic Lateral Sclerosis: A Mendelian Randomization Study. *Ann. Neurol.* 87, 434-441. doi:10.1002/ana.25671

Zhao, Q., Wang, J., Hemani, G., Bowden, J., and Small, D. S. (2020). Statistical inference in two-sample summary-data Mendelian randomization using robust adjusted profile score. *Ann. Stat.* 48, 1742-1769. doi:10.1214/19-AOS1866

Zhao, Y., Xu, Y., Wang, X., Xu, L., Chen, J., Gao, C., et al. (2020). Body Mass Index and Polycystic Ovary Syndrome: A 2-Sample Bidirectional Mendelian Randomization Study. *J. Clin. Endocrinol. Metab.* 105, dgaa125. doi:10.1210/clinem/dgaa125

Zhou, Y. C., Sun, X. B., and Zhou, M. G. (2019). Body Shape and Alzheimer's Disease: A Mendelian Randomization Analysis. *Front. Neurosci.* 13, 1084. doi:10.3389/fnins.2019.01084

Zhu, Z., Zhang, F., Hu, H., Bakshi, A., Robinson, M. R., Powell, J. E., et al. (2016). Integration of summary data from GWAS and eQTL studies predicts complex trait gene targets. *Nat. Genet.* 48, 481-487. doi:10.1038/ng.3538
